# Precipitation of binary quasicrystals along dislocations

**DOI:** 10.1038/s41467-018-03250-8

**Published:** 2018-02-23

**Authors:** Zhiqing Yang, Lifeng Zhang, Matthew F. Chisholm, Xinzhe Zhou, Hengqiang Ye, Stephen J. Pennycook

**Affiliations:** 1 Shenyang National Laboratory for Materials Science, Institute of Metal Research, Chinese Academy of Sciences, School of Materials Science and Engineering, University of Science and Technology of China, Shenyang, 110016 China; 2 Division of Materials Science and Technology, Oak Ridge National Laboratory, Oak Ridge, TN 37831 USA; 3 Department of Materials Science and Engineering, National University of Singapore, Singapore, 117576 Singapore

## Abstract

Dislocations in crystals naturally break the symmetry of the bulk, introducing local atomic configurations with symmetries such as fivefold rings. But dislocations do not usually nucleate aperiodic structure along their length. Here we demonstrate the formation of extended binary quasicrystalline precipitates with Penrose-like random-tiling structures, beginning with chemical ordering within the pentagonal structure at cores of prismatic dislocations in Mg–Zn alloys. Atomic resolution observations indicate that icosahedral chains centered along [0001] pillars of Zn interstitial atoms are formed templated by the fivefold rings at dislocation cores. They subsequently form columns of rhombic and elongated hexagonal tiles parallel to the dislocation lines. Quasicrystalline precipitates are formed by random tiling of these rhombic and hexagonal tiles. Such precipitation may impact dislocation glide and alloy strength.

## Abstract (web summary)

Quasicrystal precipitation in alloys should be favored at dislocations, which break crystal symmetry, but almost all precipitates at dislocations are periodic. Here, the authors show rare quasicrystal formation along dislocations in a magnesium zinc alloy with atomic resolution.

## Introduction

Dislocations are linear defects that break the local symmetry of bulk crystal lattices. They carry atomic shear steps during plastic deformation of crystals and thereby enable the formability of crystalline materials^1^. Importantly, dislocations in crystalline materials can also facilitate heterogeneous precipitation, prior to homogeneous precipitation in the perfect matrix^2–7^. Structure of the precipitates is determined by chemical composition of the materials, transformation temperatures, and holding time, according to the Gibbs isotherm^8^. Precipitates at dislocations have been found with crystal structures the same as those within regions away from dislocations in various crystalline materials since the 1950s^2–6,9^. It seems that localized occurrence of forbidden symmetries at dislocation cores has had little impact on precipitate structures.

But interestingly, icosahedral clusters consisting of a small amount of atoms which show fivefold rotations have low free energy, and can exist in liquid metals^10–12^. And quasicrystals can form based on icosahedral clusters present in undercooled liquid during solidification^13–18^. It is also known that fivefold rings of atoms may be produced at dislocation cores in crystals^1,19^. This knowledge about quasicrystals and dislocations^1,13–18,20^ implies that dislocations in crystals might serve as nucleation sites for icosahedral clusters. However, it has been unclear whether icosahedral clusters form and interact with the broken symmetry along dislocations in crystalline materials at the earliest stage of precipitation^2–5^.

Here, we report precipitation of quasicrystals showing Penrose-like random-tiling structures along dislocations in Mg–Zn alloys, based on atomic resolution high-angle annular dark field (HAADF) scanning transmission electron microscopy (STEM) investigations. We believe this to be the first case of a binary quasicrystalline phase formed by dislocation-assisted precipitation since the solidified Cd-Yb and Cd-Ca alloys were first reported in 2000^21,22^.

## Results

### Zn segregation and atomic structure of dislocation cores

**Fig. 1:**
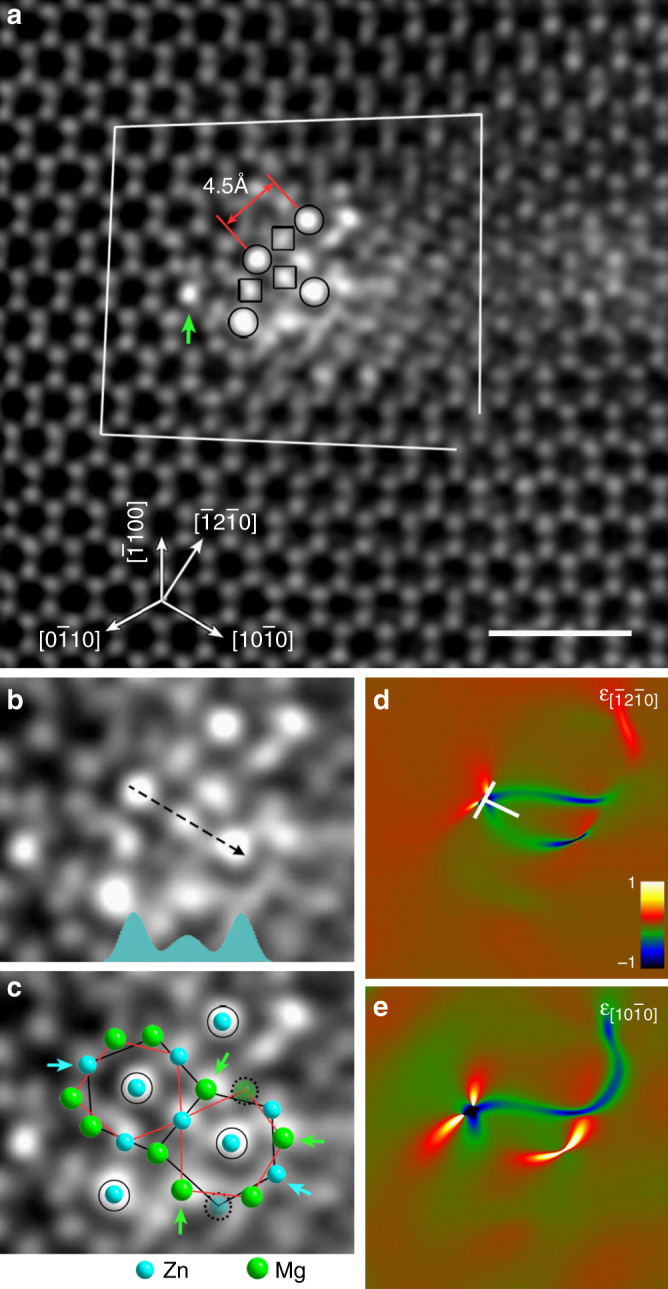
Zn segregation forming incomplete icosahedral chains at dislocations. **a** HAADF-STEM image. **b**, **c** Enlarged images of the dislocation core region shown in **a**. The intensity profile at the bottom of **b** indicates that the amount of octahedral Zn atoms is twice that of the middle column. Laves MgZn_2_ icosahedra are superimposed on **c**. Those atomic columns indicated by arrows are still mostly occupied by anti-site Zn or Mg atoms, and the two columns circled in dots are not yet occupied. **d**, **e** Lattice strain around the dislocation (⊥) measured along $[\bar 12\bar 10]$ and $[10\bar 10]$ directions, indicating presence of a dislocation nearby the upper left icosahedral chain shown in **b**. The scale bar in **a** represents 1 nm

Figure 1a shows a high-resolution HAADF-STEM image recorded along $[0001]_{\mathrm{Mg}}$ from a region with brighter intensity peaks owing to Zn segregation, which is observed in cold-rolled samples after annealing at 573 K for ~ 5 min. Those brighter spots are distributed along a prismatic edge dislocation with a Burgers vector of 〈**a**〉 = $1/3\langle 11\bar 20\rangle$, according to the closure failure of the Burgers circuit. Furthermore, the four atomic columns enriched with Zn indicated by circles are quite close to octahedral interstices near the dislocation core region, and their intensity is about twice those indicated by squares (Fig. 1b).

Careful analysis shows that there are 10 atomic columns around some of the octahedral Zn atomic columns, forming icosahedral chains^10,23^, as shown in Fig. 1c. These icosahedral chains are still under-developed chemically and structurally, compared with those in the MgZn_2_ Laves phase. Juxtaposed packing of icosahedral chains commonly exists in Frank–Kasper intermetallic compounds, such as Laves and μ phases^24,25^. The spacing between neighboring octahedral Zn columns is ~ 4.54 ± 0.04 Å (Fig. 1a), matching well the distance of 4.53 Å between neighboring icosahedral chains in MgZn_2_ C14 Laves phase (one stable crystal with lattice parameters, *a* = 5.233 Å, *c* = 8.566 Å). Because the observed Zn segregation along the dislocation shown in Fig. 1a does not form complete unit cells of the C14 Laves phase yet, we refer to it as a Cottrell atmosphere with partial ordering, rather than a precipitate. Zn segregation and partial ordering modify the dislocation core structure (Supplementary Fig. 1), and introduce additional lattice distortion around the dislocation as revealed by geometrical phase analysis^26^ (Fig. 1d, e).

### Tiny precipitates at dislocation cores

**Fig. 2:**
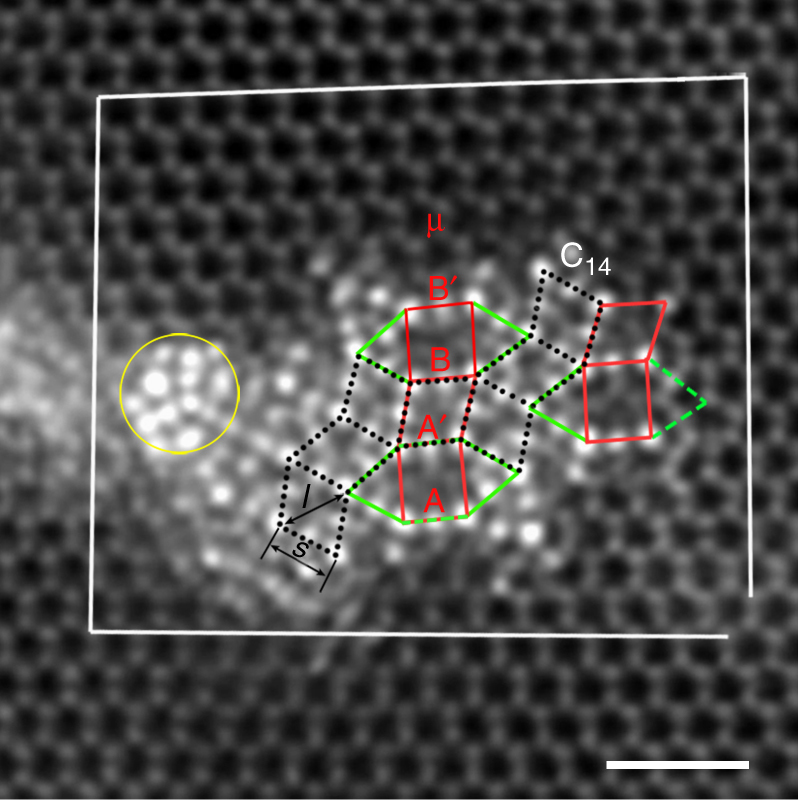
Complex precipitate along a dislocation. This precipitate is composed of rhombic and elongated hexagonal tiles. Neighboring icosahedral chains in C14 Laves and μ phases have two different separations: a shorter one, 4.53 Å (referred to as *s*) for face-sharing pairs, and a longer one, 5.25 Å (referred to as *l*) for edge-sharing pairs. The length of the longer sides of rectangles is *l*. The scale bar represents 1 nm

Figure 2 shows a high-resolution HAADF-STEM image for an edge 〈**a**〉 dislocation associated with more icosahedral chains, present commonly in cold-rolled samples after annealing at 573 K, and samples processed by friction stir processing. Interestingly, further segregation of Zn atoms did not bring about a C14 Laves MgZn_2_ precipitate, although unit-cell-scale slabs can be identified, as indicated by zigzag-stacked dotted rhombic tiles. Four icosahedral chains may also form a rectangular tile. One rhombus and a rectangle form a sub-unit of one μ unit-cell^25^. One μ rectangular tile and two icosahedral chains outside the longer sides of the rectangle together form an elongated hexagonal tile. Therefore, this precipitate is composed of rhombic and elongated hexagonal tiles. In addition, icosahedral chains with a higher level of Zn segregation exist at the region indicated by a yellow circle in Fig. 2. This kind of high segregation along dislocations was occasionally observed alone (Supplementary Fig. 2).

### Large quasicrystalline precipitates at dislocation cores

**Fig. 3:**
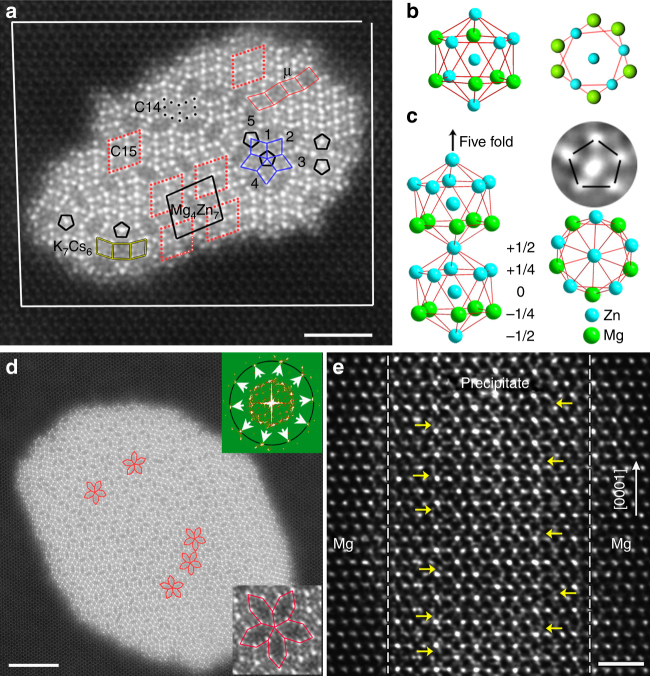
Precipitates showing Penrose-like random-tiling structures. **a** HAADF-STEM image recorded along [0001]_Mg_ for a precipitate in samples annealed at 573 K. **b** An icosahedron in Laves MgZn_2_. **c** An atomic model for chemically ordered icosahedral chains. **d** Atomic structures of typical precipitate in samples annealed at 613 K for 60 min. **e** HAADF-STEM image showing atomic structures of a precipitate recorded along $\langle 11\bar 20\rangle _{\mathrm{Mg}}$. Atoms out of basal planes are at positions close to octahedral interstices, e.g., those indicated by yellow arrows in **e**. The scale bars in **a**, **d** and **e** represent 2, 5 and 1 nm, respectively

Figure 3a shows a larger precipitate with an even more complex structure associated with an 〈**a**〉 dislocation. Besides unit-cell-scale slabs of C14 Laves and μ phases, tiny domains showing C15 Laves, Mg_4_Zn_7_ and K_7_Cs_6_ structures coexist in this precipitate, leading to five different orientations of both tiles. Interestingly, the five rhombuses outlined by blue lines form a domain showing fivefold symmetry. Peripheral atoms of this icosahedral chain are arranged with equal separation on a regular pentagon. And there are five, rather than four Zn columns surrounding the central Zn column of this icosahedral chain. Therefore, this icosahedral chain is chemically ordered along the fivefold axis, as shown in Fig. 3c. Icosahedral chains with Zn pentagons appear randomly in this precipitate, as seen in Fig. 3a. Furthermore, elongated hexagonal tiles are also distributed randomly with five different orientations numbered 1 to 5 in Fig. 3a. This arrangement of rhombic and elongated hexagonal tiles is similar to Penrose-like (Supplementary Fig. 3) random tiling in quasicrystals^13,14,23,27^. Interestingly, the Penrose-like random-tiling structure could be retained with further growth of precipitates by annealing at 613 K for 60 min (Fig. 3d). Five elongated hexagonal tiles may form a domain with a chemically ordered icosahedral chain (Fig. 3c) and showing fivefold symmetry, as highlighted in red in the bottom-right inset in Fig. 3d. In addition, there is a high density of chemically ordered icosahedral chains being shared by different arrangements of rhombic and elongated hexagonal tiles (Supplementary Figs. 4 and 5) in the precipitate shown in Fig. 3d. The upper-right inset in Fig. 3d is a Fourier diffractogram showing 10 reflections distributing on a ring, as indicated by arrows. This is to say that quasicrystalline precipitates with Penrose-like random-tiling structure (Fig. 3), instead of crystals with translational ordering, are obtained finally, starting from Zn segregation and the formation of icosahedral clusters at dislocations (Figs. 1 and 2). The structural stability of quasicrystalline precipitates should be associated with the higher entropy of Penrose-like random-tiling structures^27–32^, compared with periodic packing of icosahedral chains in a Bravais lattice.

Figure 3e shows an atomic resolution Z-contrast image recorded along the $\langle 11\bar 20\rangle _{\mathrm{Mg}}$ direction for a small precipitate, demonstrating preferential growth of quasicrystals along the $[0001]_{\mathrm{Mg}}$ direction (Supplementary Fig. 6). There are many intensity spots at or close to octahedral interstitial sites, as indicated by yellow arrows in Fig. 3e. No translational symmetry or chemical ordering can be identified for the precipitate in planes normal to $[0001]_{\mathrm{Mg}}$, according to Fig. 3e. This is owing to the nature of Penrose-like random tiling of rhombic and elongated hexagonal tiles composed of icosahedral chains in the precipitate.

### Simulation of the prismatic 〈a〉 dislocation in Mg

**Fig. 4:**
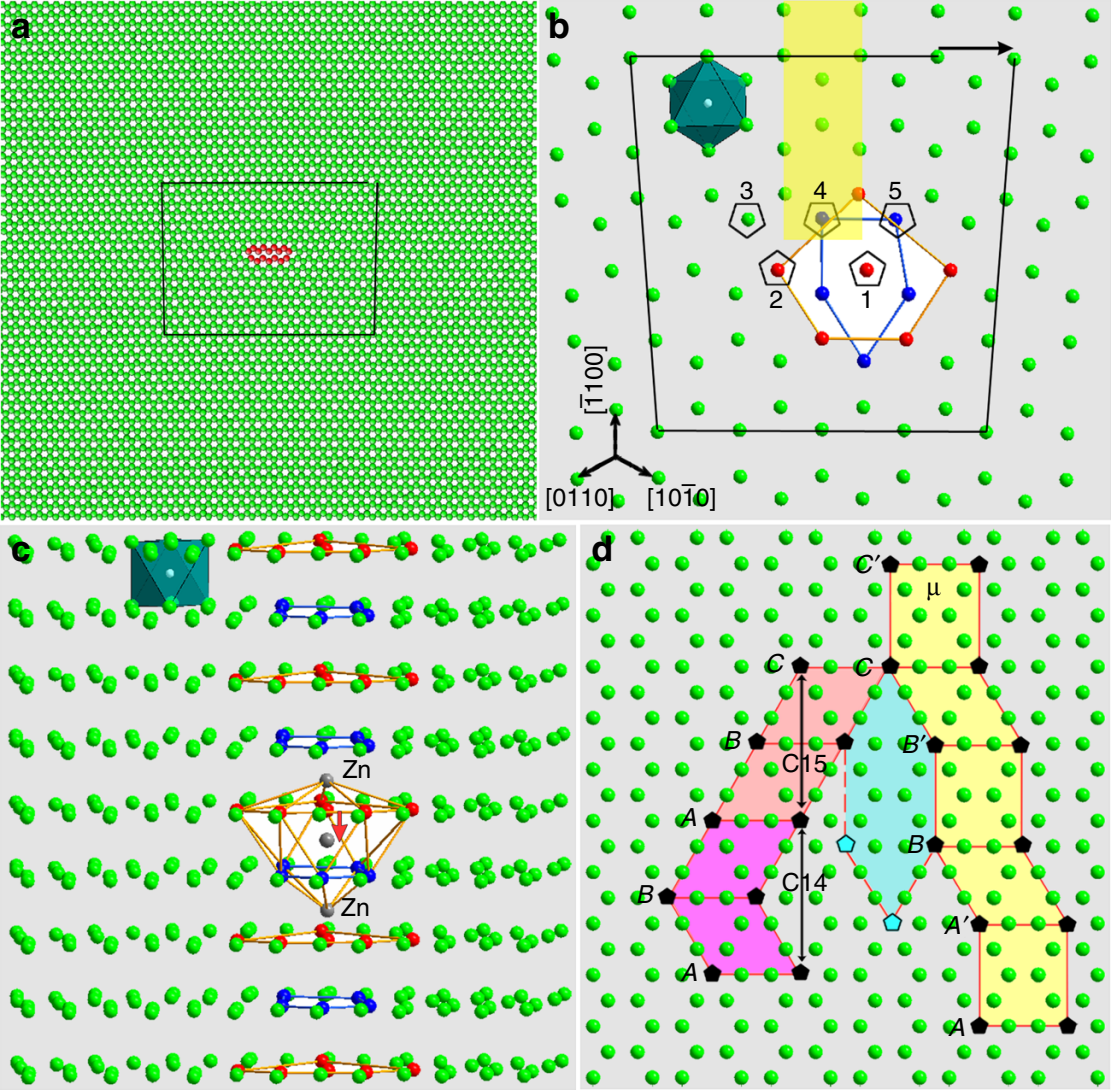
Atomic model for the formation of an icosahedron and arrangements of icosahedral chains at prismatic edge dislocations in Mg. **a** The [0001] projection for MD simulation of a prismatic 〈**a**〉 edge dislocation in Mg. Some atoms at the dislocation core are shown in red. **b** Enlargement of the dislocation core region showing anti-symmetrical pentagonal chains at the dislocation core viewed along [0001]. Atoms belonging to the extra half atomic plane are indicated by the yellow rectangle. There are five anti-symmetrical pentagonal chains surrounding atomic columns 1–5. The pentagonal chain surrounding atomic column 1 is highlighted. An octahedral interstice with its center at the white sphere is outlined. **c** Perspective view of the dislocation core with three Zn interstitial atoms along a direction close to $[\bar 1100]$. **d** Formation of unit cells of C14, C15, μ phases, and elongated hexagonal units through different packing of icosahedral chains represented by pentagons

We performed molecular dynamics (MD) simulations^33^ to obtain the atomic structure of prismatic dislocation cores in Mg, in order to find clues for understanding its role in the precipitation of Penrose-like random-tiling structures. Details of the simulations are given in the Methods. MD simulations show that there are basal pentagons (fivefold rings of atoms) at the core of prismatic 〈**a**〉 edge dislocations in Mg, as outlined in Fig. 4a, b and Supplementary Fig. 7. Interestingly, those icosahedral chains we observe and the pentagonal chains at dislocations in Mg are in the same direction, with their fivefold axes parallel to the dislocation lines. Pentagons on adjacent basal planes are anti-symmetrical (Fig. 4b), if we neglect their difference in size and shape, which is similar to the case of an icosahedron (Fig. 3b). Furthermore, most interatomic distances of these pentagon Mg atoms along the prismatic 〈**a**〉 edge dislocation are close to those in icosahedral chains in Mg–Zn crystalline compounds (Supplementary Fig. 8), which may facilitate the formation of icosahedral domains at dislocations in Mg. This structural information provides a basis for understanding and modeling precipitation of random-tiling quasicrystals at the dislocation cores.

### Formation of icosahedral chains along dislocations

When the local concentration of Zn atoms segregated at the dislocation reaches a sufficient level, structural transformation would occur through rearrangement of atoms^7,8,34^. An icosahedron would be formed, if three Zn atoms appeared between neighboring pentagons along [0001] direction, as shown by gray spheres in Fig. 4c. The middle Zn atom was originally at the center of the large pentagon and it moved downward by ~ 0.13 nm, as indicated by the red arrow. This hypothesis is consistent with atomic resolution observations of atoms between adjacent basal planes (Fig. 3e). The large basal pentagon would thus shrink slightly, leading to the formation of an icosahedron with distortion much smaller (Fig. 1) than that shown in Fig. 4c. Atoms in the columns at the center of pentagons at the dislocation core in Mg are out of basal planes (Supplementary Fig. 9), which would provide 50% of atoms to form a [0001] Zn pillar acting as the central atomic column of an icosahedral chain. An icosahedral chain can be formed by subsequent formation of icosahedra on top of each other by following the growing Zn pillar.

Once a tiny precipitate is formed, some Zn atoms arriving at the precipitate/Mg interfaces may occupy octahedral interstices^34^, producing [0001] Zn pillars, which can guide the formation and growth of icosahedral chains (Figs. 1a, 3e). Figure 4d shows examples for the packing of icosahedral chains to form unit-cell-scale units of C14, C15 Laves, μ phases and an elongated hexagon in Mg, without considering structural relaxation. Different tiles can be connected through sharing icosahedral chains in different orientations. The fivefold axis along the icosahedral chains makes it possible to connect neighboring tiles in five different orientations, which is responsible for the formation of Penrose-like random tiling (Figs. 2–4).

## Discussion

The structural units of the Mg–Zn quasicrystals are simple icosahedra (13 atoms), so they are much smaller than those hierarchical multi-shell clusters, such as Bergman clusters (136 atoms) and Yb–Cd clusters (158 atoms) in other quasicrystals^13,24,35^. Local one-dimensional icosahedral ordering in the Mg lattice is energetically favorable, owing to the intrinsic low energy state of an icosahedral packing of atoms^10^. It is most likely that an icosahedral chain was first formed based on the pentagonal structure at the core of a prismatic $1/3\langle 11\bar 20\rangle$ dislocation (Figs. 1 and 4). This was followed by the formation of other adjacent icosahedral chains templated by the rule of Penrose-like random tiling, as shown in Figs. 1–3.

If all tiny domains/tiles showing Penrose-like random tiling (Figs. 2 and 3) were decreased to the (sub-)unit-cell scale of the corresponding Frank–Kasper phases, translational order would disappear, leading to the formation of quasicrystals^13,36,37^. Moreover, random-tiling domains in the precipitates are still not larger than unit cells of the corresponding crystalline phases after annealing at 613 K for 60 min (Fig. 3d). The excellent stability of random-tiling quasicrystals is attributed to the maximized entropy density^28,29,31,32^. The formation of quasicrystals along dislocations is in contrast to precipitation of C14 Laves MgZn_2_ crystals in grains without dislocations in undeformed Mg–Zn samples (Supplementary Fig. 10). Therefore, pentagonal chains at dislocations should play a critical role in precipitation of Penrose-like random-tiling structures in the Mg–Zn alloys^38^.

Tiny, dissimilar precipitates in a material matrix can act as obstacles to dislocation motion, having an important role in strengthening alloys. Vickers microhardness was increased from 65.8 ± 2.8 to 87.0 ± 4.4 upon the formation of quasicrystalline precipitates (Fig. 3d) in Mg–Zn alloys after annealing at 613 K for 60 min, which means a relative increase of ~ 32% in microhardness of the alloy. This relative increase (32%) in microhardness of the present Mg–Zn alloy is similar to that (29%) for a Mg–Zn-Gd alloy with high density $\langle 0001\rangle$ rod precipitates of crystalline β′-Mg_7_Gd, although the density and volume fraction of the quasicrystalline rods of Mg–Zn is lower than that of β′-Mg_7_Gd rods (Methods, Supplementary Figs. 6 and 11).

Our atomic resolution observations provide clear demonstration of Zn segregation along dislocations with a preference for one-dimensional icosahedral ordering. Formation of icosahedral chains is initially templated by the nucleation and growth of individual atomic pillars of Zn atoms occupying octahedral interstitial sites of the Mg matrix. Penrose-like random tiling of rhombic and hexagonal tiles composed of icosahedral chains leads to quasicrystalline precipitates. This work shows how quasicrystals can nucleate and grow from the broken symmetry of a dislocation core in crystals, shedding new light on fundamental understanding of both dislocations and quasicrystals. Our results may have implications for precipitation strengthening of solids using quasicrystals, as dislocations are important lattice defects in strained alloys.

## Methods

### Materials preparation

Two Mg–Zn alloys, Mg-5.0Zn, and Mg-9.0Zn (weight %), were prepared by induction melting of high purity Mg and Zn under argon atmosphere protection. The Zn concentrations of Mg-5.0Zn and Mg-9.0Zn alloys are quite similar to commercial ZK60 Mg alloys^39–47^ and Mg–Zn-RE (RE, rare earth elements) alloys^48–56^ with icosahedral quasicrystalline strengthening phase, respectively. Intermetallic compounds in Mg–Zn alloys include MgZn_2_, Mg_4_Zn_7_, Mg_21_Zn_25_, MgZn, Mg_2_Zn_11_, and Mg_7_Zn_3_^57^. These binary Mg–Zn compounds are all crystalline materials, and none of them is a crystal approximant. No quasicrystals have been reported in Mg–Zn binary alloys prepared by solidification^58,59^. A Mg–Zn-Gd alloy with a Zn:Gd atomic ratio of ~ 1:2 was prepared as a control sample. As-cast Mg–Zn-Gd samples were first heated at 773 K for 10 h, followed by quenching into water. $\langle 0001\rangle$ rod precipitates of crystalline β′-Mg_7_Gd (Supplementary Fig. 11) were formed in the Mg matrix during hot compression at 573 K to a strain of ~ 20% with a strain rate of 2 × 10^−4^ s^−1^.

### Materials processing

The Mg-5.0Zn alloy was cold rolled with a thickness reduction of ~ 10.0% with 3.0–4.0% each route. Cold-rolled Mg-5.0Zn samples were annealed at 573 K for different time ranging from 5 to 15 min, to obtain small precipitates. In addition, the Mg-9.0Zn alloy was processed by friction stir processing with a rotation speed of 1200 rounds per minute and a moving speed of 100 mm per minute, producing an engineering strain of ~ 2000%, which is much larger than that of cold rolling process^60,61^. Besides introducing high plastic strain in the processed region of the materials, friction stir processing usually results in an instantaneous temperature raise to above 473 K, which may activate dynamic precipitation. Therefore, small precipitates were observed in samples treated by friction stir processing. The high plastic strain and temperature raise can lead to dynamic precipitation during the friction stir processing. After friction stir processing, Mg-9.0Zn samples were annealed at 613 K for 60 min, to study the structural stability and growth of precipitates formed during the friction stir processing.

### Microstructural characterization

HAADF-STEM observations were carried out on an aberration-corrected Nion UltraSTEM 100 microscope and a Titan 60-300 STEM microscope. The beam convergence half angle of the Nion microscope and Titan microscope is 30 and 25 mrad, respectively. The collection half angles of the HAADF detector of the Nion and Titan microscopes are, respectively, 86–200 mrad and 60–290 mrad; both settings are able to eliminate influence of strain contrast around defects effectively^62,63^. The HAADF detector collects electrons that pass close to the atomic nuclei and, thus, scatter with intensities that approach the *Z*^2^ (*Z* is the atomic number) dependence of Rutherford scattering, so local structural and chemical information can be obtained with atomic resolution by HAADF-STEM imaging^62,63^. The atomic number of Mg and Zn is 12 and 30, respectively. Therefore, it is possible to image a single Zn interstitial atom in Mg using HAADF-STEM technique^34^, which makes it a powerful way to investigate Zn segregation and tiny precipitates at dislocation cores. Energy dispersive X-ray spectroscopy measurements were performed to obtain local compositions of precipitates and their surrounding regions. Some of the rods showed brightness variation along their length viewed along the $\langle 11\bar 20\rangle$ zone axis, see the ones indicated by circles in Supplementary Fig. 6a. This kind of phenomenon was owing to overlap of rods at different heights in the sample, as shown in Supplementary Fig. 12a and b. No solutes other than Zn were detected within the quasicrystalline precipitates formed along dislocations in the present Mg–Zn alloys, as shown in Supplementary Fig. 12c and d.

### Microhardness measurements

Vickers microhardness tests were performed using an MVK-H300 microhardness tester equipped with a diamond pyramidal indentor. A load of 50 g was applied for 10 s for each measurement. At least six points were measured for each sample. The Vickers microhardness for Mg–Zn alloys before and after the precipitation of $\langle 0001\rangle$ rods with the Penrose-like random-tiling structure was measured to be 65.8 ± 2.8 to 87.0 ± 4.4, respectively, demonstrating a relative increase in microhardness of ~ 32% as a result of quasicrystalline precipitation. The Vickers microhardness of solution-treated Mg–Zn–Gd samples was 82.5 ± 3.7, whereas it was increased to 106.3 ± 6.3 after the precipitation of crystalline β′-Mg_7_Gd during hot compression. It should be pointed out that there is also work hardening in the Mg–Zn–Gd samples, as shown by the appearance of stacking faults with Suzuki segregation^20^ in the Mg grain (Supplementary Fig. 11). The relative increase (~ 29%) in microhardness of the Mg–Zn–Gd alloy is slightly smaller than that of the Mg–Zn alloys, although two kinds of strengthening structures are present, Mg_7_Gd precipitates and stacking faults with Suzuki segregation^20^, in the Mg–Zn–Gd alloy.

### MD simulations

The freely available open-source code LAMMPS^33^, and EAM (embedded-atom method) potential developed by Sun et al.^64^ were used to simulate the dislocation core structure in Mg. The specified energy tolerance is set as 10^−15^. The dislocation core structure simulated by MD using this potential is basically consistent with the results obtained by first-principles calculations^65^. There are 80,000 atoms in the supercell, with size 25.5 × 27.6 × 2.6 nm along $[11\bar 20]$, $[\bar 1100]$ and [0001], respectively. The dislocation line is parallel to [0001]. A prismatic edge $\langle \mathbf{a}\rangle$ dislocation was generated by displacing all atoms according to the Volterra solution. Periodic boundary conditions were used along the dislocation line (i.e., the [0001] axis), whereas the boundaries along the other two axes ($[11\bar 20]$ and $[\bar 1100]$) were fixed in the MD simulations. MD simulations were performed at three temperatures, 0, 300, and 600 K, in order to check the stability of the prismatic $\langle \mathbf{a}\rangle$ dislocation core, as shown in Supplementary Fig. 13. Both the 300 and 600 K simulations were performed for 5 ns. It was found that the fivefold rings at the dislocation core could stay stable within the time-frame of the simulations at 600 K (Supplementary Fig. 13), although it is higher than the annealing temperature (573 K) required to promote precipitation in cold-rolled Mg–Zn alloys (Figs. 1, 2 and 3a).

### Data availability

All data are available from the corresponding authors on reasonable request.

## Electronic supplementary material

Supplementary Information

Peer Review File
