# Supplementary material for: Precipitation of binary quasicrystals along dislocations
Source: Nat Commun. 2018 Feb 23;9:809. doi: 10.1038/s41467-018-03250-8 (PMC5824953; doi:10.1038/s41467-018-03250-8)
Supplement: Supplementary file 1 — Supplementary Information [file 41467_2018_3250_MOESM1_ESM.pdf]

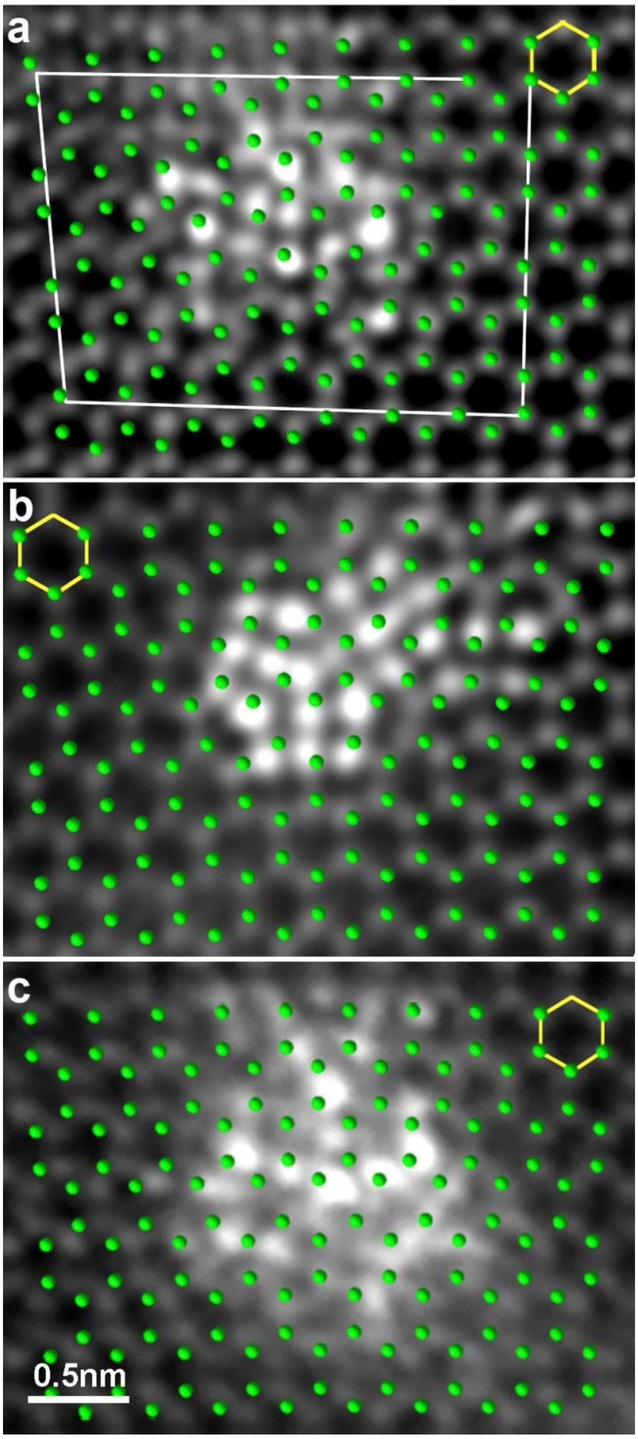

Supplementary Figure 1. Severe lattice distortion with Zn segregation at dislocations. **a-c** Comparison between three experimental images and the simulated dislocation core structure (green dots), demonstrating the structural modification and lattice distortion due to Zn segregation and partial ordering at dislocations in Mg-Zn alloys. A Burgers circuit is overlapped in (**a**), showing the presence of a prismatic ⟨**a**⟩ dislocation, as an example.

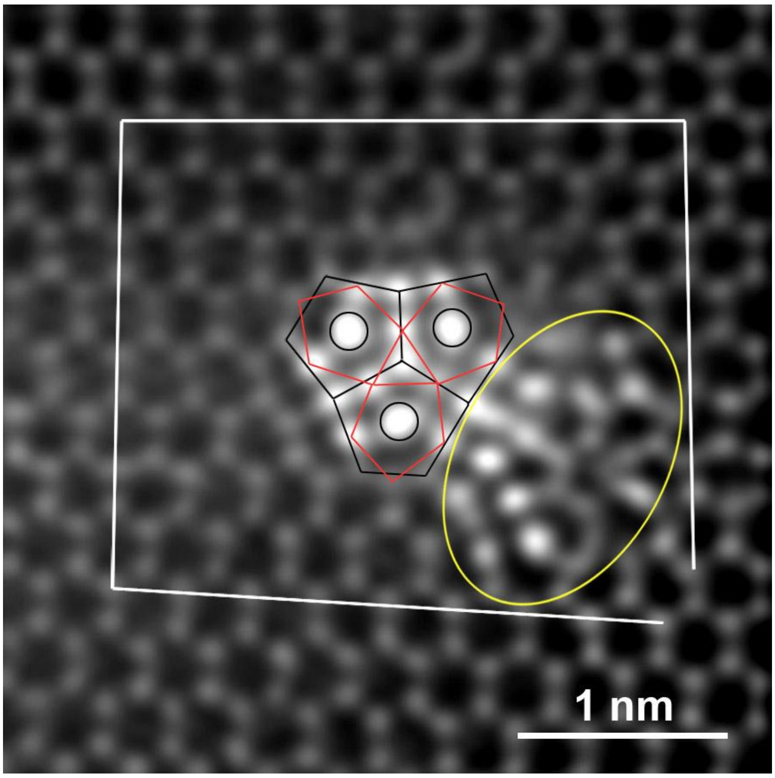

Supplementary Figure 2. High level of Zn segregation at a dislocation core region. No less than seven, instead of four or five, among the ten columns surrounding Zn columns indicated by black circles, are Zn-rich. Replacement of several Mg columns with Zn columns allows the local denser packing of icosahedral chains, since Zn is smaller than Mg. Separations between icosahedral chains are all basically equal to *s* in C14 Laves phase (~ 4.5 Å), and they share faces. This might be formed based on three neighbouring 5-fold atomic rings at a dislocation core, according to comparison with the dislocation core structure shown in Fig. 4a in the manuscript and Supplementary Fig. 7. Segregation of Zn atoms resulted in significant changes in lattice structure within the region indicated by the yellow ellipse, which could lead to formation of randomly oriented icosahedral chains like that shown in Fig. 2 in the manuscript, driven by the maximization of entropy density.

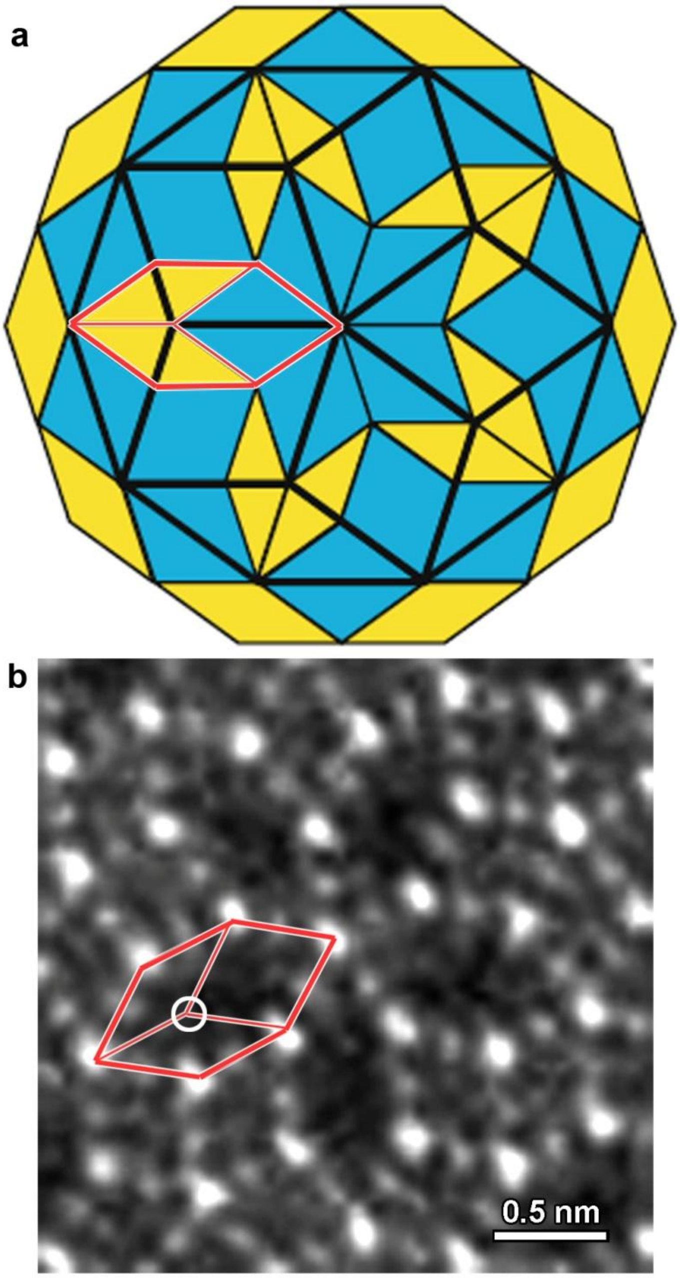

Supplementary Figure 3. Comparison between ideal Penrose tiling and our experimental tiling. **a** Penrose tiling. **b** Experimental result. One fat and two thin rhombic tiles form an elongated tile in the Penrose tiling, as outlined by red lines in (**a**). The elongated hexagonal tile overlapped on the experimental image in (**b**) has the same size as that in (**a**). Such elongated tiles are present randomly in experimentally observed precipitates, as shown in Figs. 3 in the manuscript and Supplementary Fig. 6. But, atoms at the position indicated by a white circle are Mg, instead of a central Zn column of an icosahedral chain, which are different from others at the corners of the elongated hexagonal tile. This is different from ideal Penrose random tiling. Therefore, the tiling in the experimentally observed precipitates is referred to as "**Penrose-like**" random tiling in the present study.

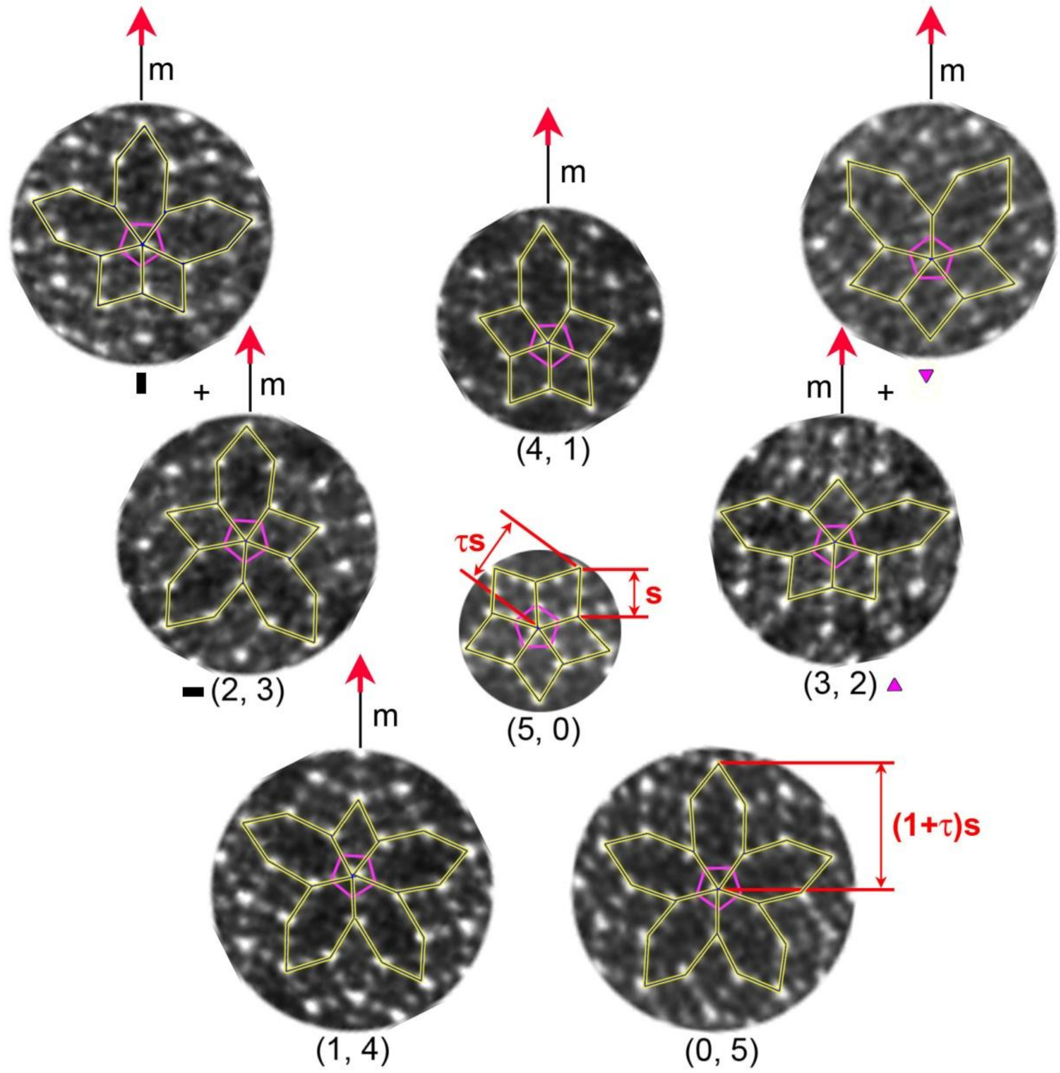

Supplementary Figure 4. Chemically ordered icosahedral chains and surrounding rhombic or elongated hexagonal tiles. One chemically ordered icosahedral chain (as indicated by the purple pentagon in each case) can be shared by n (n = 5, 4, 3, 2, 1, or 0) rhombic tiles and 5-n elongated tiles, which is indicated by (n, 5-n). There are two different arrangements of rhombic and elongated hexagonal tiles for cases of (3, 2) and (2, 3), as indicated by differently orientated purple triangles and black rectangles, respectively; while there is only one for others. There is '*one*' mirror plane for structures of (1, 4), (2, 3), (3, 2) and (4, 1), while structures (5, 0) and (0, 5) have '*five*' mirror planes. And a specific orientation of these local structures is indicated by a red arrow. The edge length of rhombic and elongated hexagonal tiles is s (s = 0.45 nm), and their longer diagonals are τs and $(1+\tau)s$, respectively. $\tau = (1+\sqrt{5})/2 \approx 1.618$ is the golden mean. The distortion of rhombic and elongated tiles is mainly from sample drift during imaging.

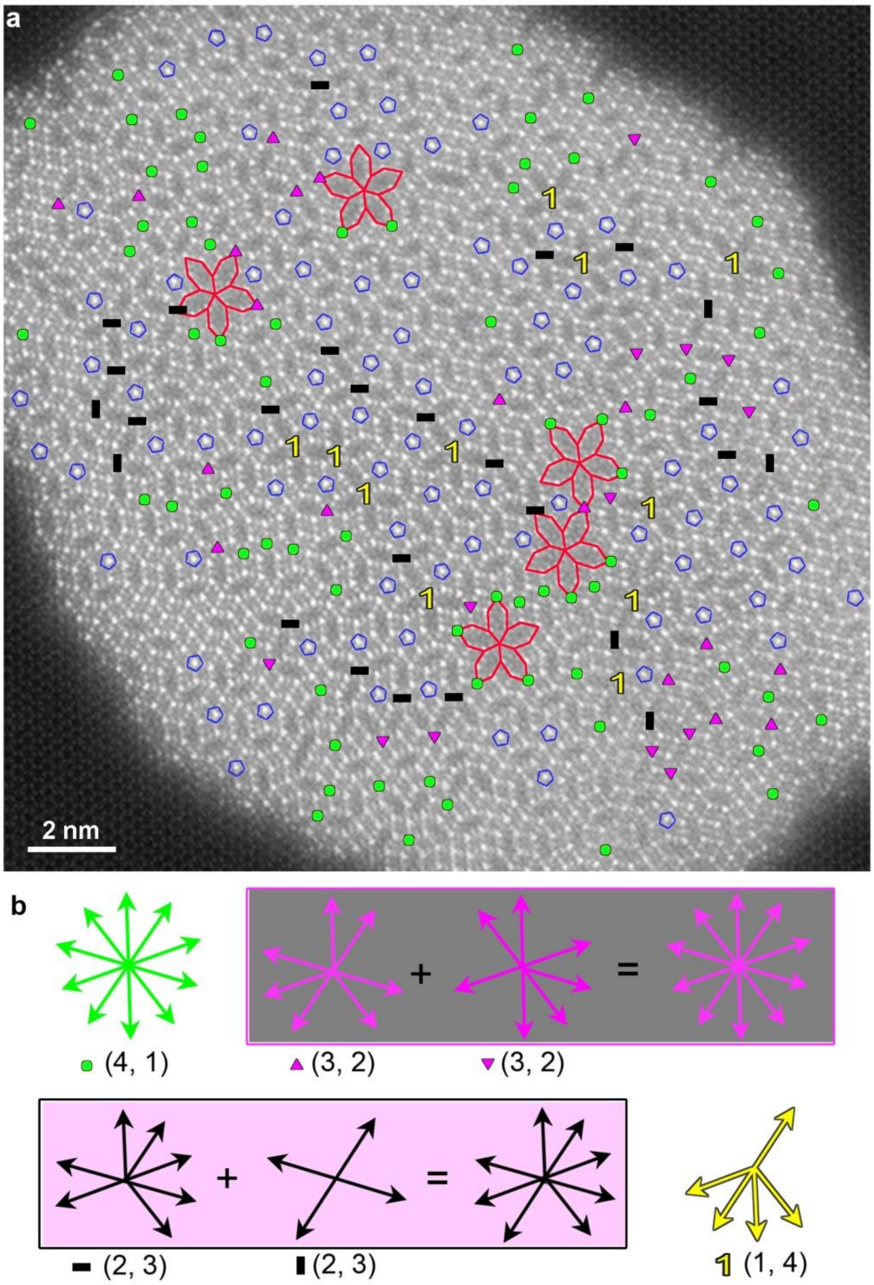

Supplementary Figure 5. Penrose-like random tiling of different tiles in a precipitate. **a** Atomic-resolution HAADF-STEM image of the precipitate shown in Fig. 3d in the manuscript. Different symbols overlapped on the image indicate random distribution of chemically ordered icosahedral chains with different mixtures of rhombic and elongated hexagonal tiles. Blue pentagons, green dots, purple triangles, black rectangles, yellow ‘**1**’ and red flowers stand for structures of (5,0), (4, 1), (3, 2), (2, 3), (1, 4) and (0, 5), respectively. Those unhighlighted icosahedral chains have either 3 of 4 nearest neighbouring icosahedral chains, and are correspondingly shared by 3 or 4 tiles, instead of 5. **b** Summary of orientations of different local structures of (4, 1), (3, 2), (2, 3) and (1, 4) in the precipitate shown in (**a**). It may be expected that there would be 10 different orientations for structures of (3, 2), (2, 3) and (1, 4), like the structure of (4, 1), if a precipitate grew large enough. The two variants for (3, 2) and (2, 3) are indicated by differently oriented symbols.

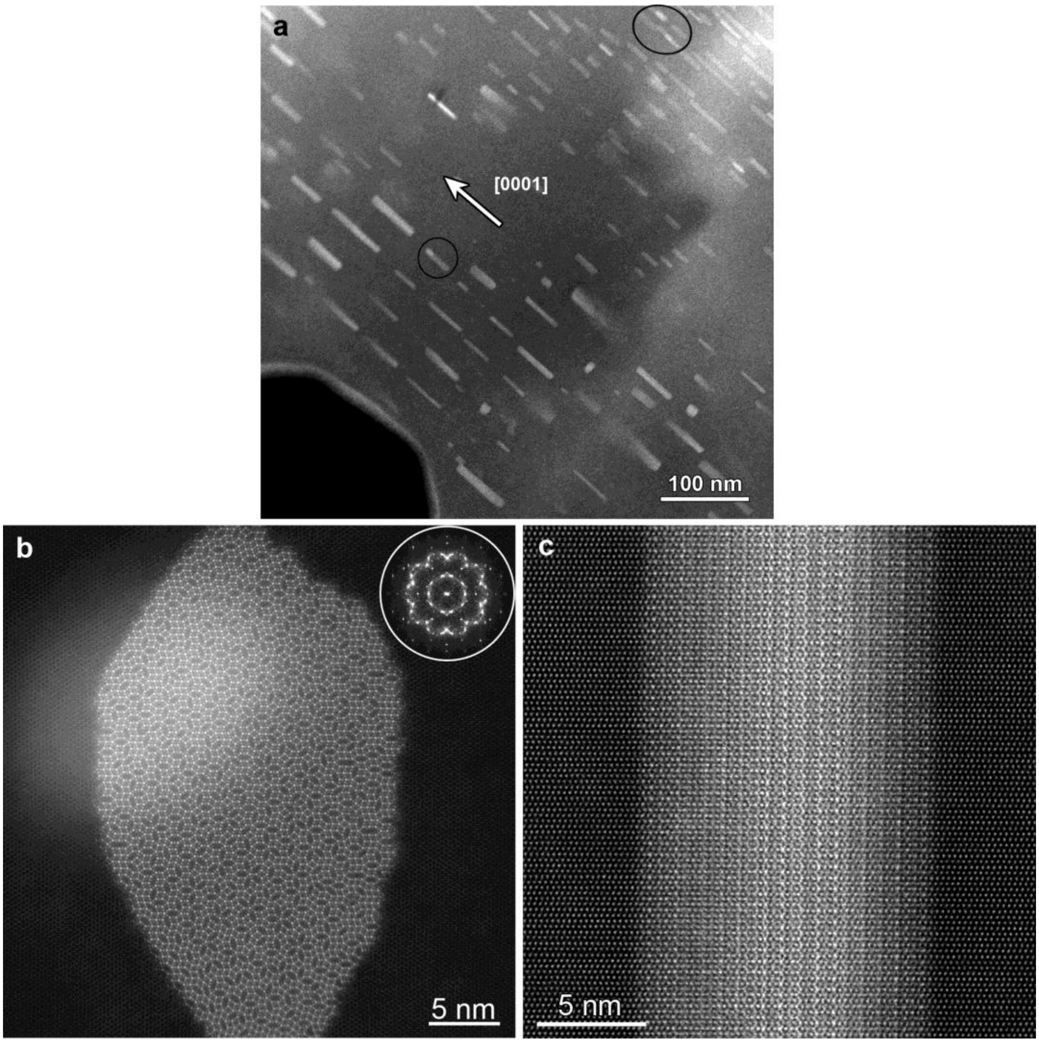

Supplementary Figure 6. Precipitates in samples annealed at 613 K for 60 min. **a** A low-magnification HAADF-STEM image showing preferential growth of nano-quasicrystals along [0001] direction of Mg. Variation in brightness along the length of the two rods indicated by circles are due to overlap of rods at different height in the sample, rather than segregation of other heavier elements (Supplementary Fig. 12 shows tilt series and EDX chemical measurements for another region). Differences in brightness of different rods are due to their different focus states, since they are at different depths in the sample. The distances between neighbouring rods range from ~36 nm to ~80 nm. **b**, **c** High-resolution HAADF-STEM images recorded along $[0001]_{Mg}$ and $\langle 11\bar{2}0\rangle_{Mg}$, respectively. The inset in (b) is a fast Fourier transformation of the image, showing ten-fold symmetry of the precipitate. Our observations demonstrate that the maximized entropy density should be enough to impede occurrence of structural and chemical translational ordering to form crystalline Frank-Kasper phases of Mg-Zn, such as C14 $MgZn_2$ or Mg_4Zn_7, at 613 K.

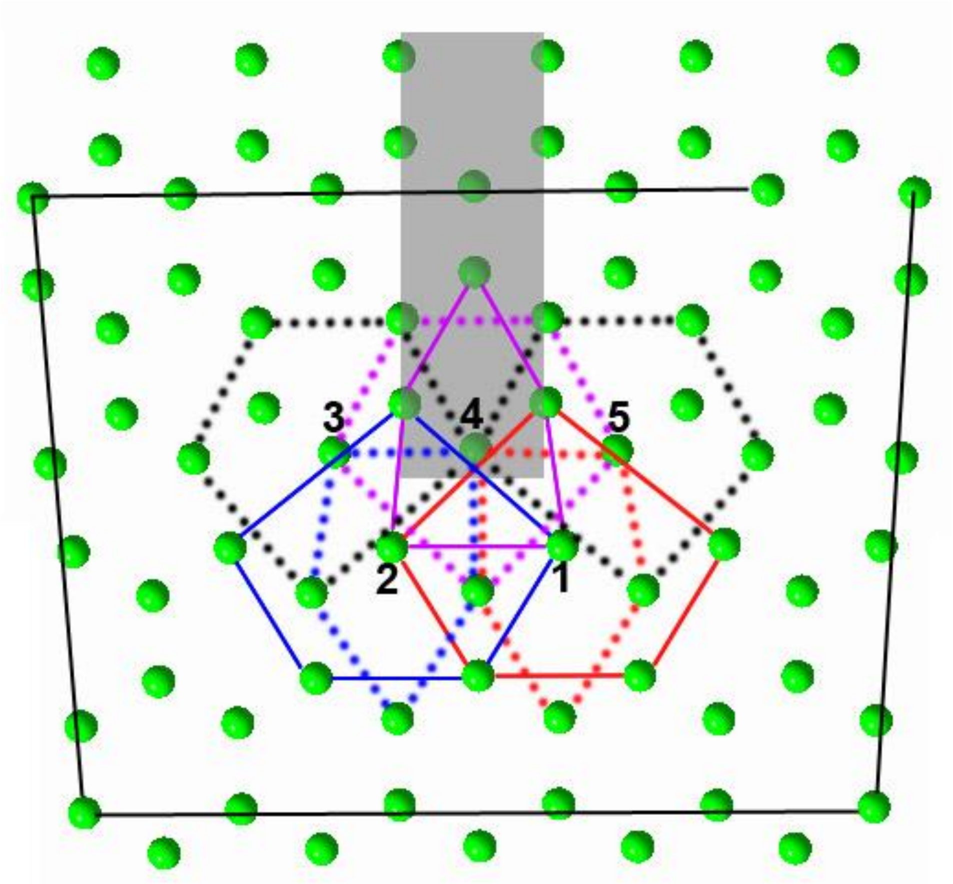

Supplementary Figure 7. Basal pentagons at an edge prismatic ⟨a⟩ dislocation in Mg. Solid and dotted lines outline pentagons in neighbouring basal planes A and B, respectively. Atoms belonging to the extra half plane of the dislocations are indicated by the gray rectangle.

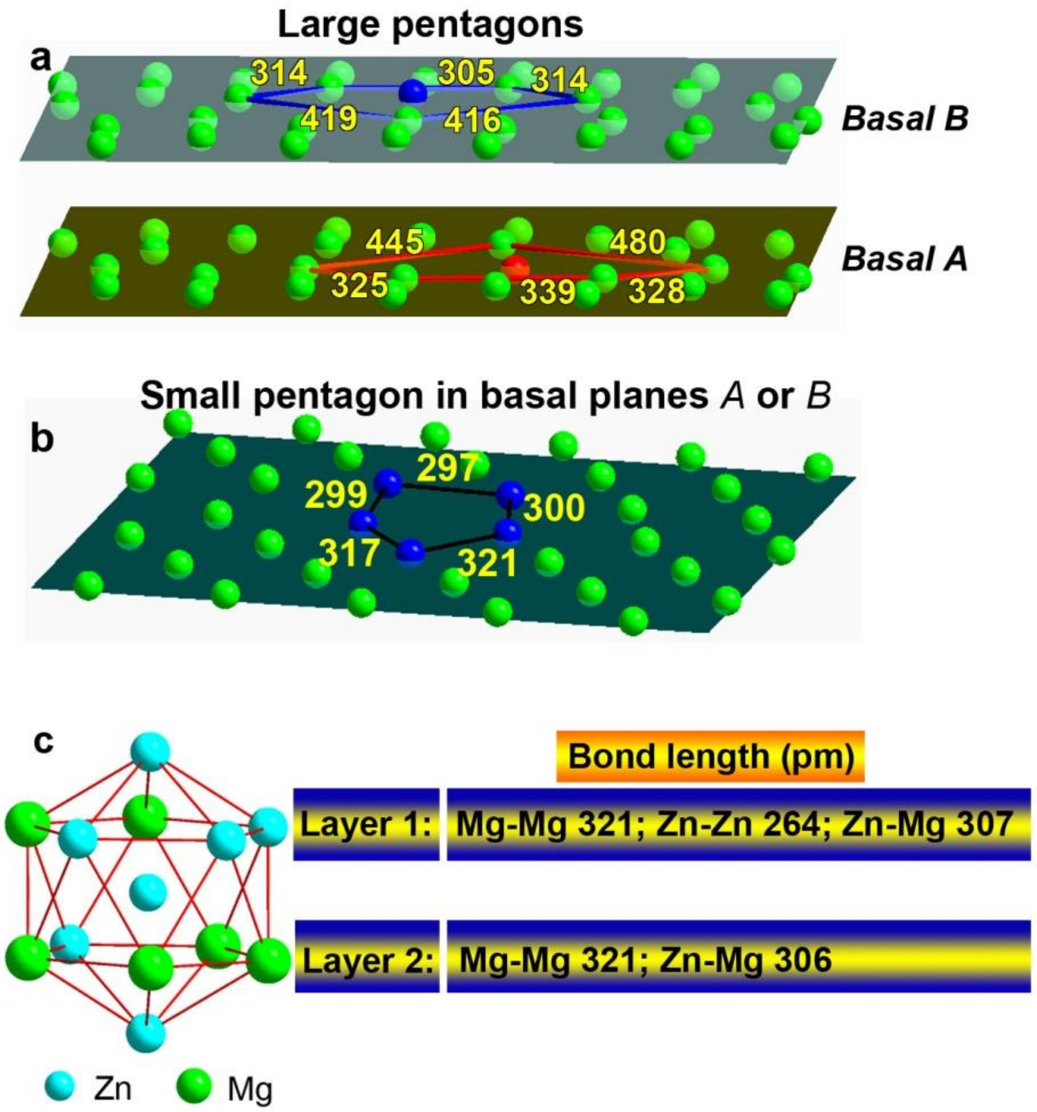

Supplementary Figure 8. Sizes of pentagons at dislocation cores. **a** Distances between Mg atoms which form large basal pentagons at an edge prismatic 〈a〉 dislocation in pure Mg. **b** One small pentagon in basal plane *A* or *B*. **c** An icosahedron and the corresponding bond length in $MgZn_2$ Laves phase. Numbers in panels (**a**) and (**b**) show interatomic distances in picometers.

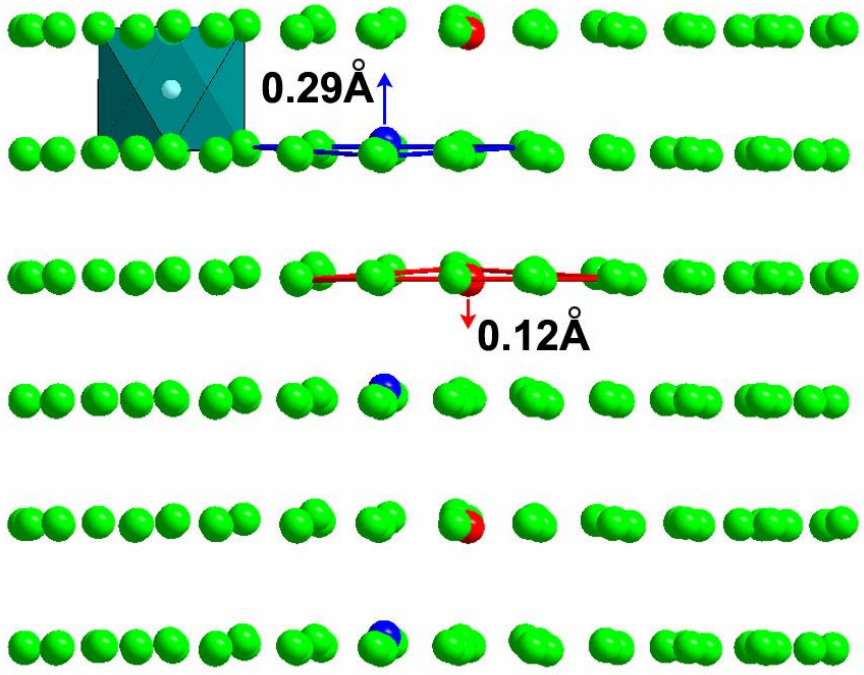

Supplementary Figure 9. Out of plane deviation of atoms at the dislocation core. The out of plane deviation of atoms is helpful for the formation of interstitials.

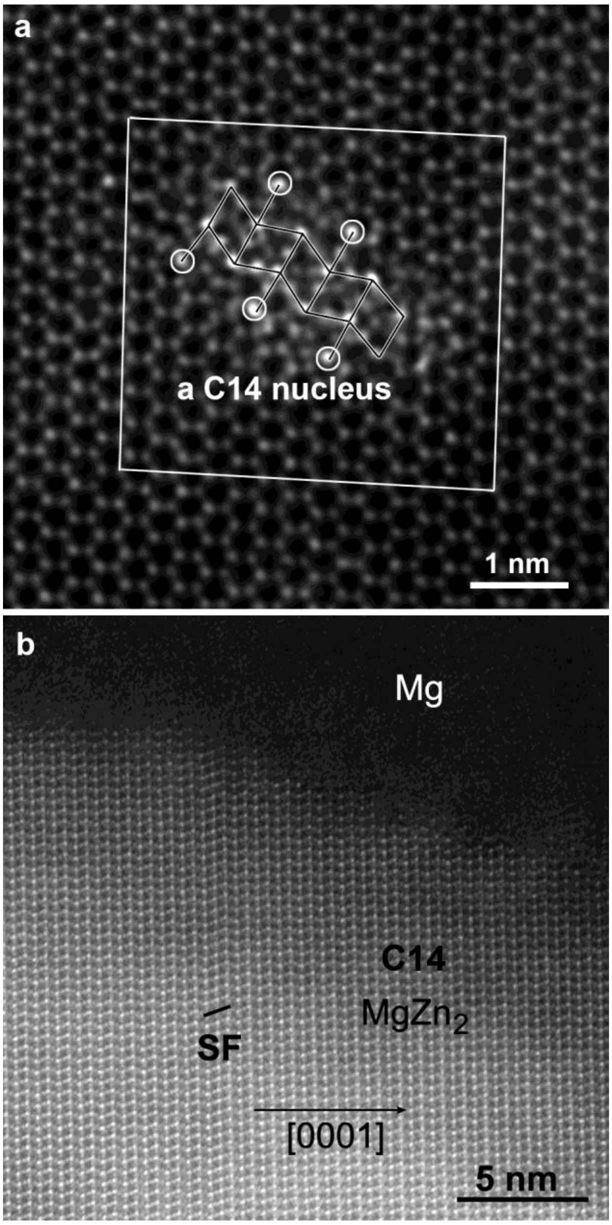

Supplementary Figure 10. Nucleation and growth of C14 Laves phase crystals in Mg grains without dislocations. **a** One tiny nucleus of C14 Laves $MgZn_2$. There is no closure failure for the Burgers circuit. Circles indicate several Zn atomic pillars formed at positions close to octahedral interstitial sites in Mg, which will guide the growth of the C14 nucleus. **b** One C14 Laves $MgZn_2$ crystalline particle precipitated in regions without dislocations in Mg grains. Basal stacking faults in C14 $MgZn_2$ are thin slices with C15 structure. The tiny C14 nucleus has its $\langle 11\bar{2}0\rangle$ zone axis parallel to [0001] of the Mg grain, but the large precipitate has no orientation relationship with the Mg grain. It would require much significant rearrangement of atoms to form an icosahedron in the Mg lattice without prismatic ⟨**a**⟩ dislocations, as shown in Supplementary Fig. 14.

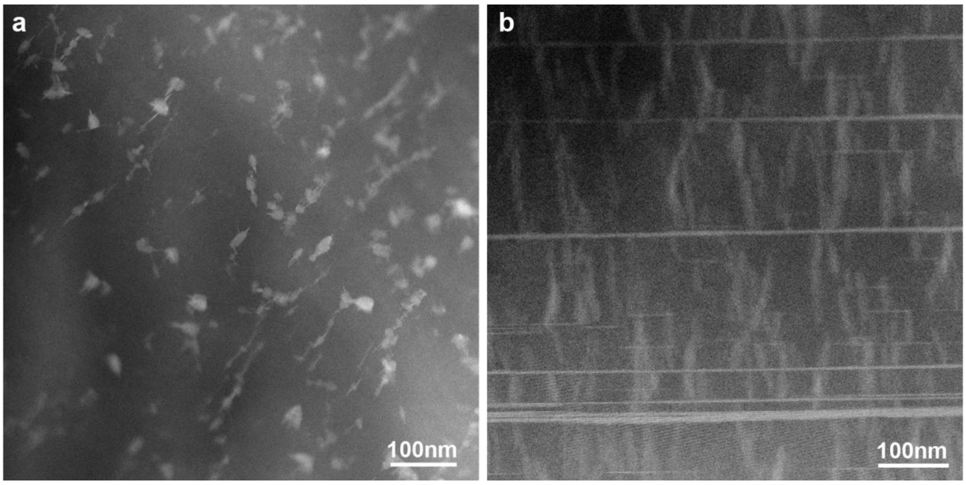

Supplementary Figure 11. Crystalline β′-Mg_7Gd rods in a Mg-Zn-Gd alloy. **a** A low-magnification HAADF-STEM image recorded along the [0001] zone axis; **b** A low-magnification HAADF-STEM image recorded along the $\langle 11\bar{2}0\rangle$ zone axis. Horizontal bright features in (**b**) are due to the presence of basal stacking faults with Zn and Gd segregation in the Mg-Zn-Gd alloy. The narrower stacking faults were produced by the dissociation of dislocations during plastic deformation, while the longer ones across the whole grain were formed during solidification. It should be pointed that those stacking faults with a relatively high density formed during plastic deformation also contribute to the microhardness, in addition to the β′-Mg_7Gd precipitates. The distances between neighbouring rods range from ~15 nm to ~37 nm. Therefore, the density of crystalline β′-Mg_7Gd precipitates in the Mg-Zn-Gd alloy is higher than that of Mg-Zn precipitates showing Penrose-like random-tiling structures in the present Mg-Zn alloy (Supplementary Fig. 6). The precipitation-induced increment of strength in the Mg-Zn-Gd alloy is estimated to be about twice that in the Mg-Zn alloy, according to the corresponding density and size of precipitates[1]. However, the precipitation-induced increment of strength is quite close (23.8 and 21.2 Hv) for the two alloys. It is seen that some precipitates in the deformed Mg-Zn-Gd sample were bent to some extent (panel **b**), implying the occurrence of plastic shear of those crystalline β′-Mg_7Gd precipitates upon interaction with basal slip in the Mg grain. Therefore, some of the small β′-Mg_7Gd precipitates could not act as effective obstacles to strengthen the alloy through the mechanism of Orowan looping for basal dislocations in Mg. It is known that no preferred shear planes exist in quasicrystals, making the very hard[2-8], so the small $\langle 0001\rangle$ rod precipitates with Penrose-like random tiling can act as effective obstacles against dislocation shear. The difference in the capability against shear deformation of different type of precipitates may be the reason for the discrepancy between experimental measurement and theoretical prediction about the precipitation-induced strengthening effect for the Mg-Zn and Mg-Zn-Gd alloys.

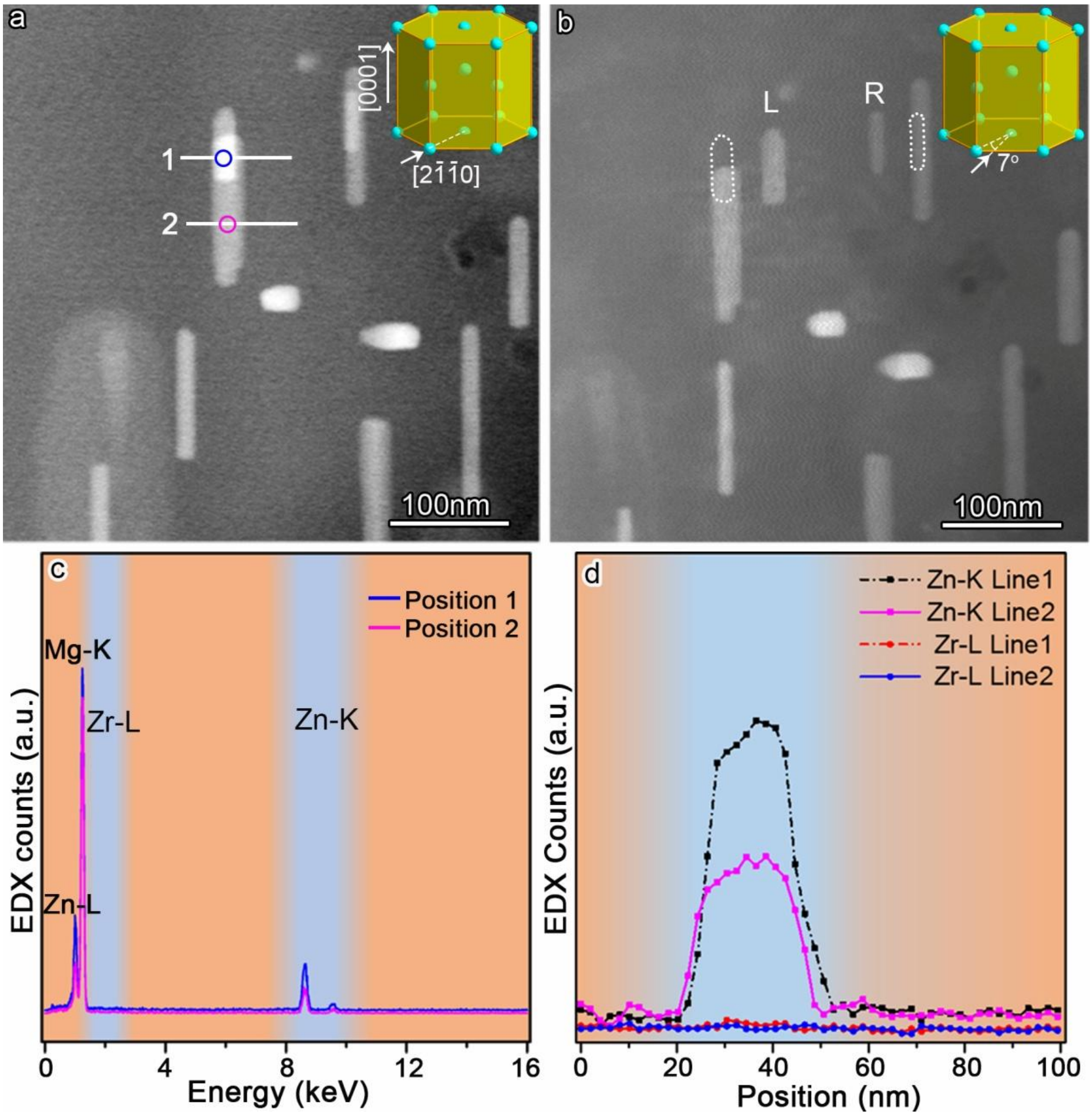

Supplementary Figure 12. Brightness variation along the length of precipitates and corresponding chemical measurements in Mg-Zn alloys. **a** A low-magnification HAADF-STEM image recorded along exactly the $\langle 11\bar{2}0\rangle$ zone axis; **b** A low-magnification HAADF-STEM image recorded after a tilt angle of about 7.0° around the [0001] axis for the region shown in (**a**), showing clearly the separation of pairs of rods; **c** EDX spectra measured from regions with different brightness, as indicated in (a); **d** Line-scan profiles along lines 1 and 2 indicated in (**a**). The shorter rod indicated by "L" is at a position above the longer one on its left, while that labeled by "R" is below the longer one on its right, judging from the rotation and their relative positions shown in (**b**), and the dotted shapes show the overlap configuration observed in (**a**) along $\langle 11\bar{2}0\rangle$. EDX measurements showed that the rods are composed of only Zn and Mg, and no signal of Zr (L peak, 2.04 keV) can be detected, as shown in (**c**). Moreover, there is no Zr in the matrix, as shown in (**d**).

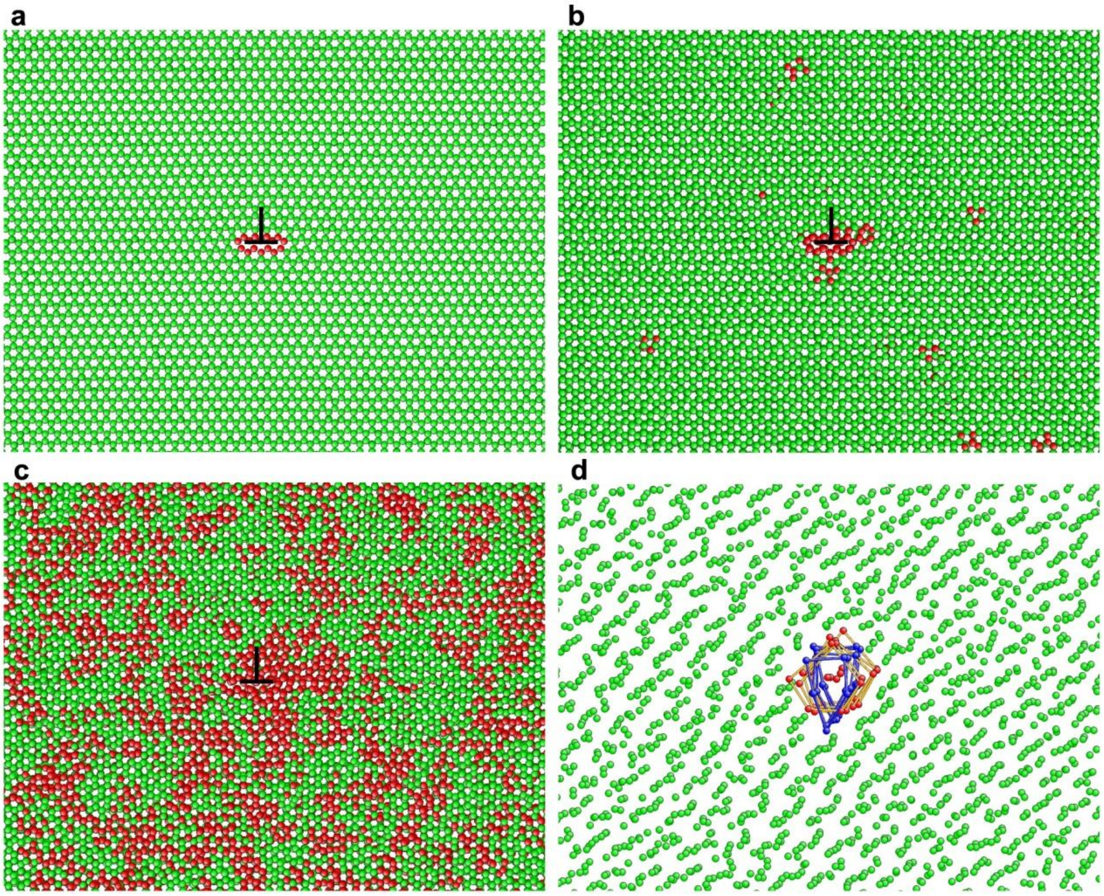

Supplementary Figure 13. MD simulations of prismatic edge $\langle \boldsymbol{a} \rangle$ dislocation cores in Mg at different temperatures. **a** 0 K; **b** 300 K; **c** 600 K. **d** A perspective view of the dislocation with one of the five-fold columns highlighted at the core derived from MD simulations at 600 K. Thermal vibration of atoms occurs throughout the lattice at 300 K and 600 K. Atoms which deviated obviously from their equilibrium HCP lattice positions due to thermal vibration are also highlighted in red in the matrix away from the dislocation core, as shown in (**b**) and (**c**). It is important to note that the five-fold atomic rings are stable when the temperature is increased to 600 K (**c** and **d**), although severe thermal vibration of atoms occurs.

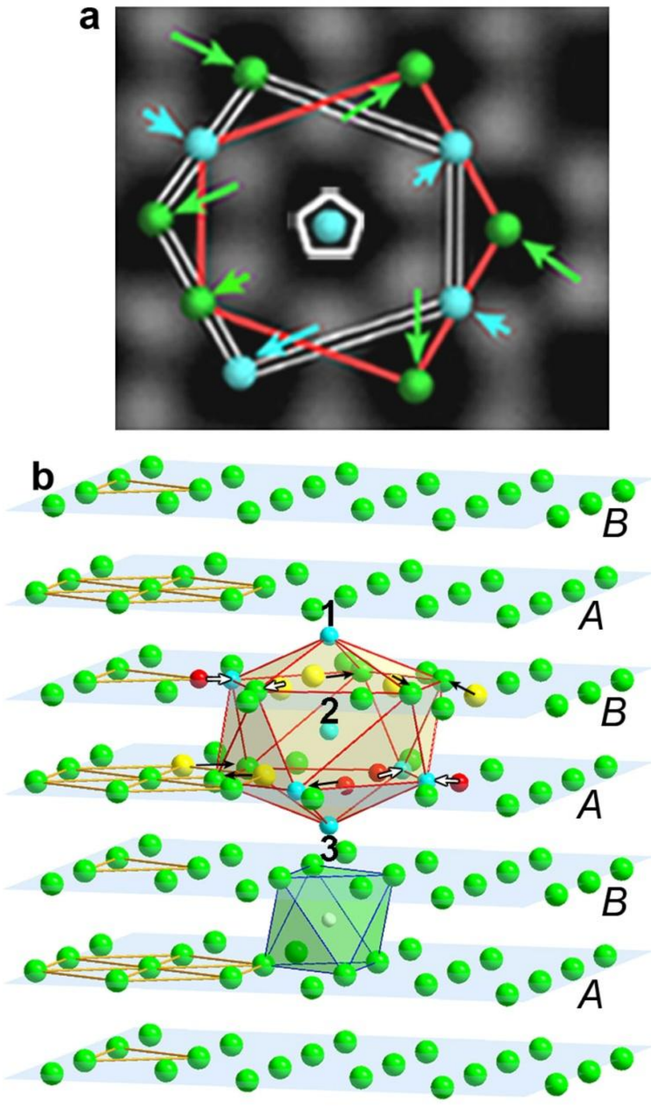

Supplementary Figure 14. Schematic model for the formation of an icosahedron in HCP Mg. **a** Projection of an icosahedral chain superimposed on a STEM image of Mg. **b** Perspective view of the formation of an icosahedron. Mg and Zn atoms are shown in green and blue, respectively. Yellow and red atoms represent Mg and Zn atoms that occupy hexagonal lattice positions, and those atoms need to shift along the arrows to icosahedral positions. The white dot indicates the center of an octahedral interstice in Mg. Atoms numbered 1, 2 and 3 are Zn octahedral interstitials.

Supplementary References

1. Gladman, T. *Mater. Sci. Tech*. **15**, 30-36 (1999).
2. Yuan, G. Y., Amiya, K., Kato, H. & Inoue, A. *J. Mater. Res*. **19**, 1531-1538 (2004).
3. Luo, Z. P., Zhang, S. Q., Tang, Y. L. & Zhao, D. S. *Scripta Metall. Mater.* **28**, 1513-1518 (1993).
4. Singh, A., Somekawa, H. & Mukai T. *Scripta Mater.* **56**, 935-938 (2007).
5. Kim, I. J., Bae, D. H. & Kim, D. H. *Mater. Sci. Eng.* A **359**, 313-318 (2003).
6. Bae, D. H., Kim, S. H., Kim, D. H. Kim, W. T. *Acta Mater.* **50**, 2343-2356 (2002).
7. Singh, A. Osawa, Y. Somekawa, H. Mukai, T., Parrish, C. J. & Shih, D. S. *Metall. Mater. Trans*. A **45**, 3232-3240 (2014).
8. Somekawa, H. Singh, A. Osawa, Y. & Mukai, T. *Mater. Trans*. **49**, 1947-1952 (2008).
