# Supplementary material for: Precipitation of binary quasicrystals along dislocations
Source: Nat Commun. 2018 Feb 23;9:809. doi: 10.1038/s41467-018-03250-8 (PMC5824953; doi:10.1038/s41467-018-03250-8)
Supplement: Supplementary file 2 — Peer Review File [file 41467_2018_3250_MOESM2_ESM.pdf]

Reviewers' Comments:

Reviewer #1:
Remarks to the Author:
The manuscript with a Reference No. NCOMM-17-23804-T reports a very fine piece of in-depth research addressing the precipitation of binary Mg-Zn quasicrystal rods along dislocations of an Mg-Zn alloy by using HAADF-STEM imaging. The evidences present in the paper are solid and convincing. Although a variety of Mg-Zn phases including <0001> rod precipitates have been observed in the Mg-Zn system, this quasicrystal Mg-Zn precipitate is a new observation made by this research. Based on in-depth atomistic structural information unveiled by HAADF-STEM and MD simulation, this research revealed the detailed mechanisms of the formation of the quasicrystal precipitates along dislocations in the alloy. Because this research did no provide any mechanical properties of this alloy, it should not speculate much about strengthening effect of the quasicrystal rods. It is likely that this quasicrystal rod precipitates just like other <0001> rod precipitates should produce very similar strengthening effect in the Mg alloy, given that <0001> rods are known to be effective in producing strengthening effect than basal precipitates in Mg alloys. In fact, a simple hardness measurement of the alloy can easily clarify easy this. Secondly, I noticed that some individual rods in extended data Fig.6a are observed with a change in brightness along their length, authors should provide explanation for the brightness variation along a single rod. Zr is frequently added in such alloys for grain refinement. Authors may check if this brightness variation along a rod is due to the presence of Zr.

Reviewer #2:
Remarks to the Author:
The manuscript reports the discovery of binary quasicrystalline patches along dislocation lines based on HAADF-STEM studies. The identified ordering could be successfully modeled by MD simulations. It is well known that pentagonal structural arrangements are quite common along grain boundaries and also around dislocation cores. What is new in the present study is the formation of of larger structural arrangements (2-3 nm?) known from quasicrystals. The results are certainly original and of interest, experiments and evaluation are done well, but I do not think that this justifies publication in Nature Communications. JAllCom, MaterTRans or a similar journal would be better suited.

Abstract l2 "local atomic configurations with forbidden symmetries" local fivefold symmmetry is not "forbidden"

The scale bar is missing in most electron micrographs.

Reviewer #3:
Remarks to the Author:
The submitted manuscript details a predominantly HRTEM investigation of a MgZr binary alloy system subjected to a deformation/temperature preparation protocol. The work finds small precipitates which have icosahedral-like structure with some larger ones having a Penrose-like random-tiling structure. The claim of the authors is that such precipitates have nucleated from prismatic dislocations within the matrix. This is argued through the idea that dislocation core structures have certain local symmetries akin to icosahedra, which may be locally deformed to produce small defect structures of a similar type as that seen experimentally. Limited simulation work is done to support this. The central experimental evidence for this assertion is derived from HRTEM images which demonstrate an incomplete Burgers vector circuit in the crystalline matrix around the observed precipitate structures.

The work is certainly interesting. Whilst not being a TEM expert, the images do seem rather good. It is not possible to judge the simulation work since the only information given is in the method section via the sentence "The freely-available open-source code LAMMPS, and EAM 360 (embedded atom method) potential were used to simulate the dislocation core structure in Mg. The specified energy tolerance is set as 10e-15." There is no information on the potential used, the stability of the structures to temperature, the simulation cell size etc. This is clearly insufficient for a journal article. Even in the main body of the text little is said - I can only assume that the simulations have demonstrated that some of the considered structures are at a local minimum and therefore structurally stable at zero Kelvin. If this is the case then even this is a trivial result, given the high symmetry of the considered structures. Much much more has to be done to give the simulations a meaningful presence to the work.

The final conclusion of the work is "This work shows how quasicrystals can nucleate and grow from the broken symmetry of a dislocation core in crystals, shedding new light on fundamental understanding of both dislocations and quasicrystals."

Given that the theoretical structures (which often have local icosahedral symmetries) can be understood in terms of topological line defects (cf. topological HCP structures, Laves and Frank-Kasper phases, Quasi-crystals, glasses through defected icosahedrally order ... there are many) the subject matter is of general interest.

However the claim that such structures are nucleated from prexisting dislocations is ultimately just a claim. An alternative scenario could be that such structures simply require such a topology to exist - that is, that they themselves can only exist as topological line defects, in the same way a dislocation does. Then the connection to the dislocation - where such precipitates nucleate at dislocations and therefore are directly connected to plasticity - as done in the paper is an assumption. More generally, is the statement that such precipitates must have a topological signature already known? Is this demanded theoretically? A central advancement of the submitted work would then be this claim and its associated observation.

One of the final statements "Our results may have implications for precipitation engineering of quasicrystals in solids, since dislocation length can be a light year per cubic meter in strained alloys, having major impact on the strength of alloys." seems entirely off-track with the reference to the distance light travels in a year.

To summarize the work is interesting and general, but not yet of the standard necessary for such a journal. The authors need to include a description of their simulation work (with a stability analysis) and also consider the above points.

Response to Reviewers' comments

Reviewer #1 (Remarks to the Author):

The manuscript with a Reference No. NCOMM-17-23804-T reports a very fine piece of in-depth research addressing the precipitation of binary Mg-Zn quasicrystal rods along dislocations of an Mg-Zn alloy by using HAADF-STEM imaging. The evidences present in the paper are solid and convincing. Although a variety of Mg-Zn phases including <0001> rod precipitates have been observed in the Mg-Zn system, this quasicrystal Mg-Zn precipitate is a new observation made by this research. Based on in-depth atomistic structural information unveiled by HAADF-STEM and MD simulation, this research revealed the detailed mechanisms of the formation of the quasicrystal precipitates along dislocations in the alloy. Because this research did not provide any mechanical properties of this alloy, it should not speculate much about strengthening effect of the quasicrystal rods. It is likely that this quasicrystal rod precipitates just like other <0001> rod precipitates should produce very similar strengthening effect in the Mg alloy, given that <0001> rods are known to be effective in producing strengthening effect than basal precipitates in Mg alloys. In fact, a simple hardness measurement of the alloy can easily clarify easy this. Secondly, I noticed that some individual rods in extended data Fig.6a are observed with a change in brightness along their length, authors should provide explanation for the brightness variation along a single rod. Zr is frequently added in such alloys for grain refinement. Authors may check if this brightness variation along a rod is due to the presence of Zr.

Reply:

(1) ***It is a very good suggestion to add microhardness results.*** Microhardness was measured and the results were added in the revised manuscript (Please see pages 9-11 and 13). ***In addition, we prepared control samples of Mg-Zn-Gd which was strengthened by crystalline [0001] rods of*** $\boldsymbol{Mg_3Gd}$. It was found that the relative increased in microhardness of Mg-Zn samples with quasicrystalline rods was

higher, compared with Mg-Zn-Gd samples with [0001] rods of crystalline Mg_3Gd. Therefore, the quasicrystalline rods of Mg-Zn have excellent strengthening effect.

(2) The variation of brightness along some rods was due to overlap of rods at different depth, instead of local segregation of other heavy elements, like Zr, as shown in the Figure below. This was confirmed by tilting the sample and direct chemical measurements. We measured the chemical composition using nano-beam energy dispersive X-ray spectroscopy, demonstrating that those rods were composed of only Zn and Mg, without Zr.

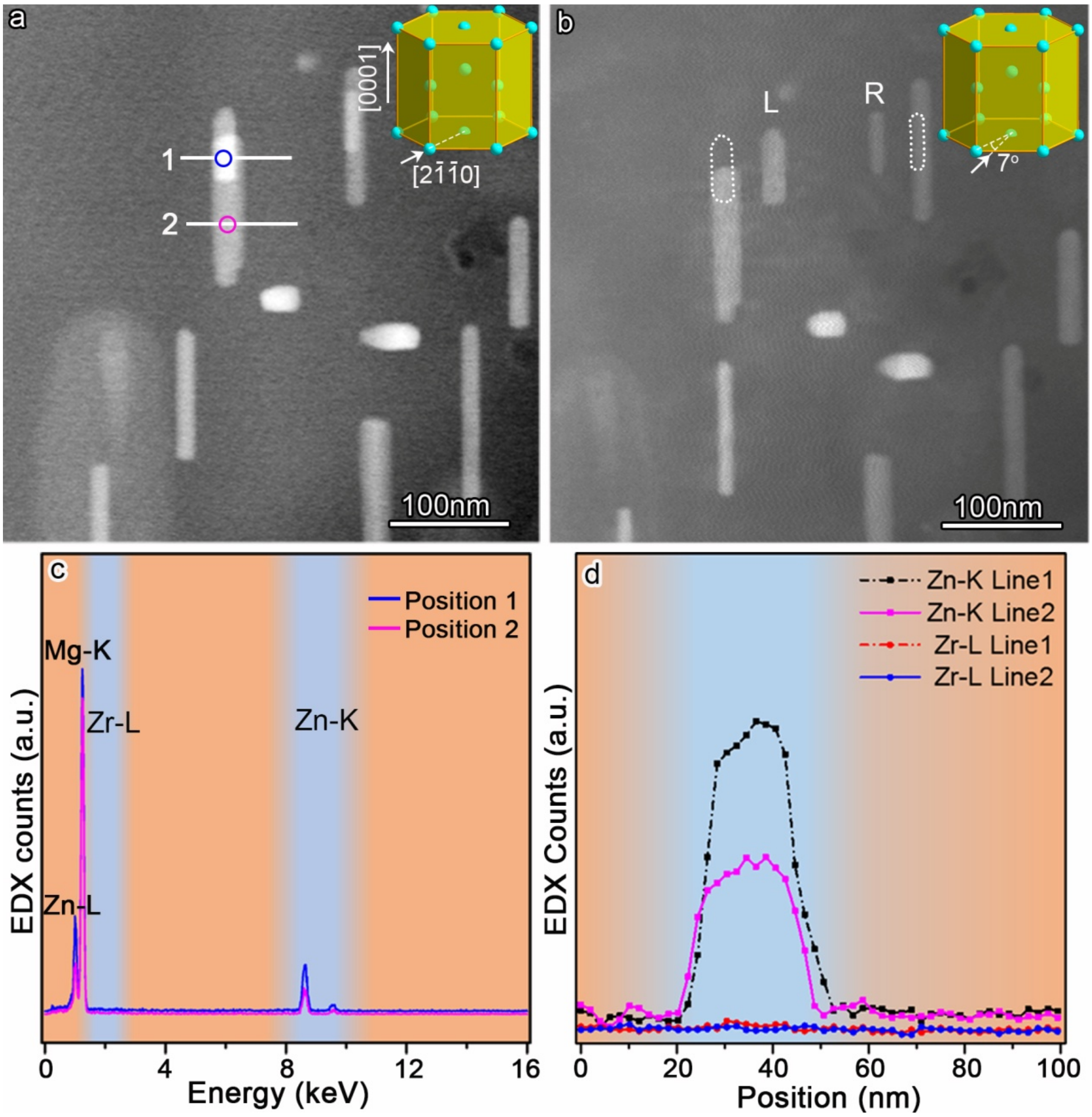

By the way, the above figure was included in the Supplementary Information (Supplementary Fig. 12).

Reviewer #2 (Remarks to the Author):

The manuscript reports the discovery of binary quasicrystalline patches along dislocation lines based on HAADF-STEM studies. The identified ordering could be successfully modeled by MD simulations. It is well known that pentagonal structural arrangements are quite common along grain boundaries and also around dislocation cores. What is new in the present study is the formation of larger structural arrangements (2-3 nm?) known from quasicrystals. The results are certainly original and of interest, experiments and evaluation are done well, but I do not think that this justifies publication in Nature Communications. JAllCom, MaterTRans or a similar journal would be better suited.

Reply: First, binary quasicrystals are rare. Secondly, not only are there no crystalline approximants in binary Mg-Zn alloys, also the structural units of the binary Zn-Mg quasicrystals are simple icosahedra composed of only 13 atoms which are much less than those large ones in other quasicrystals (such as, Bergman clusters, 136 atoms; Yb-Cd clusters, 158 atoms). Thirdly, we demonstrated that the presence of five-fold rings at dislocation cores played an important role in the precipitation of binary Mg-Zn quasicrystals. Therefore, we believe that our finding provides new knowledge for fundamental understanding of both dislocations and quasicrystals, which should be of high scientific interest.

Abstract l2 "local atomic configurations with forbidden symmetries" local fivefold symmetry is not "forbidden"

Reply: This word was deleted in the revised manuscript.

The scale bar is missing in most electron micrographs.

Reply: Scale bars in Supplementary Figs. 1, 3 and 5 were added in the revised manuscript.

Reviewer #3 (Remarks to the Author):

The submitted manuscript details a predominantly HRTEM investigation of a MgZn binary alloy system subjected to a deformation/temperature preparation protocol. The work finds small precipitates which have icosahedral-like structure with some larger ones having a Penrose-like random-tiling structure. The claim of the authors is that such precipitates have nucleated from prismatic dislocations within the matrix. This is argued through the idea that dislocation core structures have certain local symmetries akin to icosahedra, which may be locally deformed to produce small defect structures of a similar type as that seen experimentally. Limited simulation work is done to support this. The central experimental evidence for this assertion is derived from HRTEM images which demonstrate an incomplete Burgers vector circuit in the crystalline matrix around the observed precipitate structures.

The work is certainly interesting. Whilst not being a TEM expert, the images do seem rather good. It is not possible to judge the simulation work since the only information given is in the method section via the sentence "The freely-available open-source code LAMMPS, and EAM (embedded atom method) potential were used to simulate the dislocation core structure in Mg. The specified energy tolerance is set as 10e-15." There is no information on the potential used, the stability of the structures to temperature, the simulation cell size etc. This is clearly insufficient for a journal article. Even in the main body of the text little is said - I can only assume that the simulations have demonstrated that some of the considered structures are at a local minimum and therefore structurally stable at zero Kelvin. If this is the case then even this is a trivial result, given the high symmetry of the considered structures. Much much more has to be done to give the simulations a meaningful presence to the work.

Reply: (**1**) Details of the MD simulations, such as the amount (80,000) of atoms and three-dimensional size (25.5 nm × 27.6 nm × 2.6 nm) of the supercell, as well as boundary conditions were added in the revised manuscript (Methods, Page 13). By the way, a reference about the EAM potential was added (ref. 64). (**2**) We did simulations

at three different temperatures, 0 K, 300 K and 600 K. The dislocation core structure was found stable even at 600 K, irrespective of the thermal vibration of atoms. These results were added in the revised manuscript (Methods, Supplementary Fig. 13). By the way, we put Supplementary Fig. 13 below.

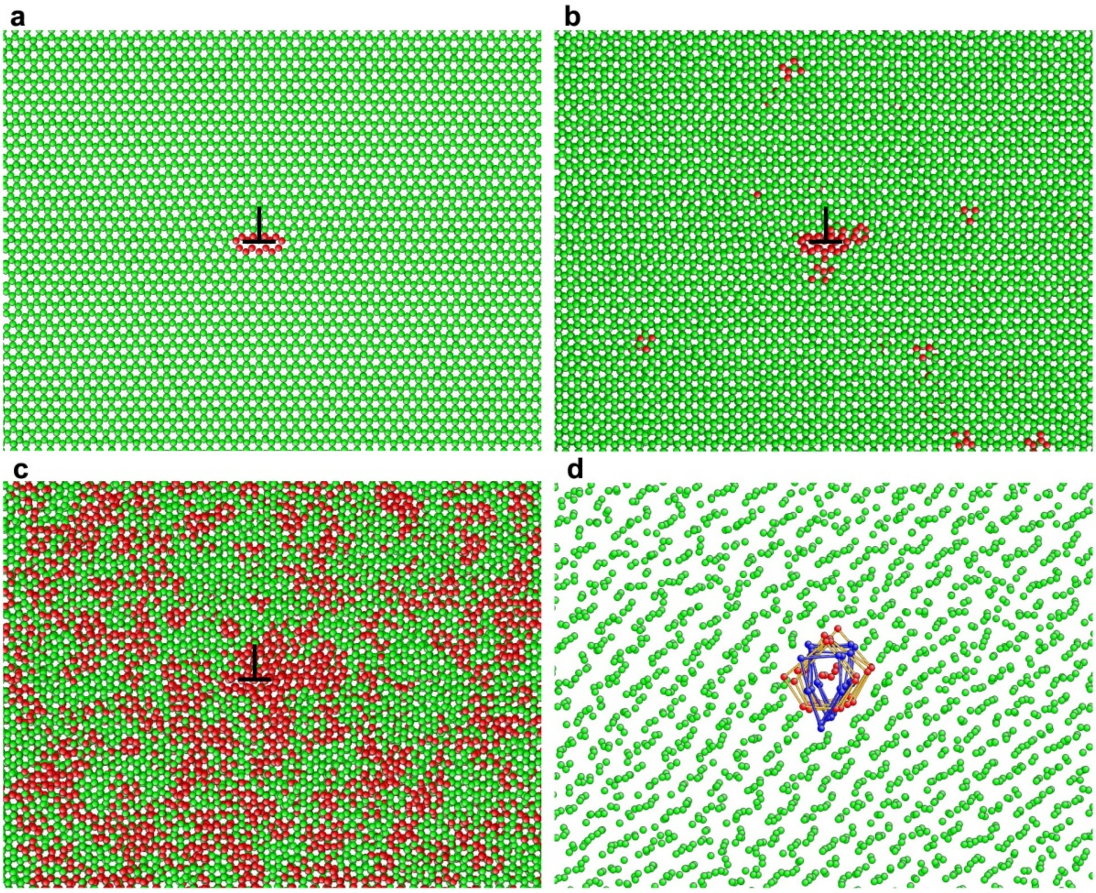

Supplementary Figure 13. MD simulations of prismatic edge <a> dislocation cores in Mg at different temperatures. **a** 0 K; **b** 300 K; **c** 600 K. **d** A perspective view of the dislocation with one of the five-fold columns highlighted at the core derived from MD simulations at 600 K. Thermal vibration of atoms occurs throughout the lattice at 300 K and 600 K. Atoms which deviated obviously from their equilibrium HCP lattice positions due to thermal vibration are also highlighted in red in the matrix away from the dislocation core, as shown in (**b**) and (**c**). It is important to note that the five-fold atomic rings are stable when the temperature is increased to 600 K (**c** and **d**), although severe thermal vibration of atoms occurs.

The final conclusion of the work is "This work shows how quasicrystals can nucleate and grow from the broken symmetry of a dislocation core in crystals, shedding new light on fundamental understanding of both dislocations and quasicrystals."

Given that the theoretical structures (which often have local icosahedral symmetries)

can be understood in terms of topological line defects (cf. topological HCP structures, Laves and Frank-Kasper phases, Quasi-crystals, glasses through defected icosahedrally order ... there are many) the subject matter is of general interest.

However the claim that such structures are nucleated from prexisting dislocations is ultimately just a claim. An alternative scenario could be that such structures simply require such a topology to exist - that is, that they themselves can only exist as topological line defects, in the same way a dislocation does. Then the connection to the dislocation - where such precipitates nucleate at dislocations and therefore are directly connected to plasticity - as done in the paper is an assumption. More generally, is the statement that such precipitates must have a topological signature already known? Is this demanded theoretically? A central advancement of the submitted work would then be this claim and its associated observation.

Reply:

(1) Quasicrystals are rare in binary alloys. In the Mg-Zn system, crystalline Mg_4Zn_7 or C14 Laves $MgZn_2$ are usually formed during solidification. It was found that the tiny nuclei showed crystalline structures, if there was not a dislocation in the Mg-Zn alloy (Supplementary Fig. 11a). Furthermore, we observed the formation of C14 Laves $MgZn_2$ within regions without dislocations through in situ TEM studies (Supplementary Fig. 11b). Therefore, we believe that the presence of dislocations should play an important role in the formation of quasicrystals in the Mg-Zn alloys.

(2) Quasicrystalline alloys are usually formed in ternary systems, such as Mg-Zn-Y, Mg-Zn-Gd. The presence of dislocations is not necessary anymore for the precipitation of ternary quasicrystals. The presence of elements with 3 valence electrons plays an critical role in those ternary alloys.

(3) All those ternary quasicrystals have crystalline approximants. The crystalline approximants are composed of clusters with a point symmetrical elements of 5-fold rotation. The Mg-Zn crystalline compounds can be described as 3-dimensional packing of distorted icosahedra consisting of 13 atoms. So, the presence of building units or clusters with a (pseudo-)5-fold rotation point

symmetrical element should be necessary for the formation of quasicrystals with 5- or 10-fold rotational symmetry.

One of the final statements "Our results may have implications for precipitation engineering of quasicrystals in solids, since dislocation length can be a light year per cubic meter in strained alloys, having major impact on the strength of alloys." seems entirely off-track with the reference to the distance light travels in a year.

Reply: This sentence was revised as: “Our results may have implications for precipitation strengthening of solids using quasicrystals, since dislocations are important lattice defects in strained alloys.”

To summarize the work is interesting and general, but not yet of the standard necessary for such a journal. The authors need to include a description of their simulation work (with a stability analysis) and also consider the above points.

Reply: Details of simulations were added and all other comments have be addressed carefully.

Reviewers' Comments:

Reviewer #1:
Remarks to the Author:
Although some changes have been made in this revised manuscript with a reference No. NCOMM-17-23804, it is disappointing that some fundamental errors remain in the current version. The strength of this manuscript is the fine microscopy investigation in revealing quasicrystal precipitation and understanding its formation mechanism. The weakness of this work is the exaggeration of the significance of the quasicrystal precipitates in strengthening the Mg-Zn alloy and some misleading statements as a result. In my opinion, the experimental evidences presented in the last paragraph in page 9 failed to support author's conclusion i.e. [0001] quasicrystalline rods have an excellent strengthening effect in Mg-Zn alloys. In fact, the hardness results demonstrate that the [0001] quasicrystalline rods, just like all other non-shearable [0001] rods, provide the same strengthening effect. Given the experimental measurement errors of ±4.4/2.8 HV in their hardness data, the errors of the relative increase in microhardness would be ±6.7/4.2%. Therefore, the 3% difference between 32% and 29% observed between quasicrystalline ppts and crystalline ppts is statistically insignificant. This means that unlike what is claimed by the manuscript, quasicrystalline rods and crystalline rods in the two alloys produced no difference in strengthening effect. If we look at the absolute hardness increases of 21.2 and 23.8 Hv for the two alloys (as stated in page 13), these values again failed in supporting the authors conclusion. In fact, if precipitates (no mater quasicrystalline or crystalline) are not able to be sheared by a dislocation, Orowan looping becomes favourable. The yield strength increases can be given by $0.84(1.2Gb/(2\pi L))\ln(x/2b)$. Clearly, the strength increase does not depend on the crystal structure of the precipitates! As result, the last sentence in the abstract and similar statement in the texts and conclusions have to be removed from the manuscript to avoid misleading their readers.

Reviewer #3:
Remarks to the Author:
I am largely satisfied with the response of the authors and, subject to the following condition, recommend publication. The authors again do not state the full information of the simulation. How many nano-seconds were simulated at the finite temperatures? Such information must be included. They should also modify the sentence in the methods section: "It was found that the five-fold rings at the dislocation core could stay stable at 600 K" to "It was found that the five-fold rings at the dislocation core could stay stable within the time-frame of the simulations at 600 K".

Response to Reviewers

Reviewer #1

Although some changes have been made in this revised manuscript with a reference No. NCOMM-17-23804, it is disappointing that some fundamental errors remain in the current version. The strength of this manuscript is the fine microscopy investigation in revealing quasicrystal precipitation and understanding its formation mechanism. The weakness of this work is the exaggeration of the significance of the quasicrystal precipitates in strengthening the Mg-Zn alloy and some misleading statements as a result. In my opinion, the experimental evidences presented in the last paragraph in page 9 failed to support author's conclusion i.e. [0001] quasicrystalline rods have an excellent strengthening effect in Mg-Zn alloys. In fact, the hardness results demonstrate that the [0001] quasicrystalline rods, just like all other non-shearable [0001] rods, provide the same strengthening effect. Given the experimental measurement errors of 4.4/2.8 HV in their hardness data, the errors of the relative increase in micro-hardness would be 6.7/4.2%. Therefore, the 3% difference between 32% and 29% observed between quasicrystalline ppts and crystalline ppts is statistically insignificant. This means that unlike what is claimed by the manuscript, quasicrystalline rods and crystalline rods in the two alloys produced no difference in strengthening effect. If we look at the absolute hardness increases of 21.2 and 23.8 Hv for the two alloys (as stated in page 13), these values again failed in supporting the authors conclusion. In fact, if precipitates (no mater quasicrystalline or crystalline) are not able to be sheared by a dislocation, Orowan looping becomes favourable. The yield strength increases can be given by $0.84(1.2Gb/(2\pi L))\ln(x/2b)$. Clearly, the strength increase does not depend on the crystal structure of the precipitates! As result, the last sentence in the abstract and similar statement in the texts and conclusions have to be removed from the manuscript to avoid misleading their readers.

Reply: We would like to thank the reviewer for raising this critical issue. We agree with the reviewer about the strengthening effect of quasicrystalline rods in Mg-Zn

based alloys. It is true that the relative increase in micro-hardness of about 32% due to the precipitation of rods with Penrose-like random tiling structures in our Mg-Zn alloys is similar to that (29%) in a control Mg-Zn-Gd sample with crystalline Mg_7Gd precipitates. We measured the distances between neighbouring rods in both samples. The distances between neighbouring quasicrystalline rods in our Mg-Zn range from ~36nm to ~80nm, while the distances between neighbouring crystalline precipitates are in the range of 15-35 nm in the Mg-Zn-Gd alloy. So, if only the Orowan looping mechanism was considered in strengthening ($\Delta\tau = 0.84(1.2Gb/(2\pi L))\ln(x/2b)$), the precipitation-induced increment of strength in the Mg-Zn-Gd alloy would be about twice that in the Mg-Zn alloy, according to the corresponding density and size of precipitates (Supplementary Figs. 6 and 11). However, the precipitation-induced increment of strength is quite close to each other (23.8 and 21.2 Hv). This suggests that some kind of softening should occur in the control Mg-Zn-Gd sample with small crystalline precipitates. It was seen that some crystalline precipitates in the deformed Mg-Zn-Gd sample were bent obviously (Supplementary Fig. 11b), implying the occurrence of plastic shear of those crystalline β′-Mg_7Gd precipitates upon interaction with basal slip in the Mg grain, indicating that some of the small β′-Mg_7Gd precipitates could not act as effective obstacles to strengthen the alloy through the mechanism of Orowan looping for basal dislocations in Mg. It is known that no preferred shear planes exist in quasicrystals, so the small ⟨0001⟩ rod precipitates with Penrose-like random tiling can act as effective obstacles against dislocation shear. The difference in the capability against shear deformation of different type of precipitates may be the reason for the discrepancy between experimental measurement and theoretical prediction about the precipitation-induced strengthening effect for the Mg-Zn and Mg-Zn-Gd alloys. Therefore, we would like to keep a brief paragraph about the mechanical properties in the manuscript (page 9). Please see below:

Tiny, dissimilar precipitates in a material matrix can act as obstacles to dislocation motion, playing an important role in strengthening alloys. Vickers microhardness was increased from 65.8 ± 2.8 to 87.0 ± 4.4 upon the formation of

quasicrystalline precipitates (Fig. 3d) in Mg-Zn alloys after annealing at 613 K for 60 minutes, which means a relative increase of ~32% in microhardness of the alloy. This relative increase (32%) in microhardness of the present Mg-Zn alloy is similar to that (29%) for a Mg-Zn-Gd alloy with high density $\langle 0001 \rangle$ rod precipitates of crystalline β′-Mg_7Gd, although the density and volume fraction of the quasicrystalline rods of Mg-Zn is lower than that of β′-Mg_7Gd rods (Methods, Supplementary Figs. 6 and 11).

Reviewer #2

(1) *I am largely satisfied with the response of the authors and, subject to the following condition, recommend publication. The authors again do not state the full information of the simulation. How many nano-seconds were simulated at the finite temperatures? Such information must be included.*

Reply: We thank the reviewer for this comments. The following sentence was added in the methods section in revised manuscript.

"Both the 300K and 600K simulations were performed for 5 ns." Please see page 14.

(2) *They should also modify the sentence in the methods section: "It was found that the five-fold rings at the dislocation core could stay stable at 600 K" to "It was found that the five-fold rings at the dislocation core could stay stable within the time-frame of the simulations at 600 K"*."

Reply: Done. Please see page 14.
